# Transcriptional responses of mouse proximal colon and colonoids during early whipworm infection

**DOI:** 10.1128/mbio.02176-25

**Published:** 2025-09-11

**Authors:** Hyeim Jung, Joseph F. Urban, Bruce A. Rosa, Makedonka Mitreva

**Affiliations:** 1 Department of Internal Medicine, Washington University School of Medicine 12275 , St. Louis, Missouri, USA; 2 United States Department of Agriculture, Agricultural Research Service, Beltsville Agricultural Research Center, Animal Parasite Diseases Laboratory and Beltsville Human Nutrition Research Center, Diet, Genomics and Immunology Laboratory 57604, Beltsville, Maryland, USA; 3 McDonnell Genome Institute, Washington University School of Medicine 12275 , St. Louis, Missouri, USA; Tsinghua University, Beijing, China

**Keywords:** *Trichuris muris*, whipworm, transcriptomics, first-stage larvae, early infection, organoids, host-parasite interactions

## Abstract

Trichuriasis, caused by the parasitic nematode *Trichuris trichiura*, affects 429–508 million people worldwide. Although the early phase of whipworm infection is crucial for establishing infection, its underlying molecular mechanisms remain unclear. In this study, we examined and compared host transcriptional responses during early infection of the mouse whipworm, *T. muris*, using first-stage larvae (L1) in proximal colons from *in vivo* models (B6 and STAT6-deficient mice) and *in vitro* colonoid models. Differentially expressed genes and functional enrichment analysis revealed that while the “neurotransmitter release” pathway was uniquely upregulated in B6 mice, the “lipid metabolism” pathway was commonly modulated in both mice and colonoids, which may have implications for intestinal epithelial function during infection. Enrichment of alternative splicing (AS) events in splicing-related pathways across all models highlighted the need for further investigation into AS regulation mechanisms and its functional roles during early infection. Temporal transcriptomic profiling of L1 *T. muris* in colonoids identified six clusters representing fundamental molecular pathways associated with parasite development and adaptation. Utilizing dual-RNA sequencing from infected colonoids, we conducted host–parasite co-expression analysis, identifying correlated gene pairs, including a negative correlation between *T. muris*-secreted serine proteases and mouse genes involved in metabolism and epithelial cell functions. These findings provide valuable insights into the dynamic transcriptional regulation during early L1 *T. muris* infection *in vivo* and *in vitro*, offering a resource for comparative studies in whipworm infection models. In conclusion, this comparative study serves as a starting point for deeper investigation into molecular mechanisms underlying early whipworm infection and opens up new opportunities for exploring host–parasite interactions.

**IMPORTANCE:**

Trichuriasis, caused by the parasitic nematode *Trichuris trichiura*, remains a major public health concern, particularly in resource-limited regions. Current anthelmintics show suboptimal efficacy against whipworm infections, highlighting the critical need for novel therapeutic strategies. This study provides a comparative framework by integrating transcriptional profiles from *in vivo* and *in vitro* models during the early infection phase of *T. muris*, a mouse model for *T. trichiura*. Through this approach, we demonstrate the potential of proximal colonoids as a model for investigating key aspects of host–parasite interactions, including epithelial invasion and transcriptional dynamics, during early *T. muris* infection. By employing dual-RNA sequencing, we not only characterize temporal gene expression dynamics of first-stage larvae but also identify host–parasite co-expression profiles, thereby shedding light on molecular pathways that may underlie infection establishment and host responses. This work builds upon and solidifies previous findings about the utility of organoid models for investigating early whipworm infection while providing a foundational resource for exploring intervention strategies targeting the initial stages of infection.

## INTRODUCTION

Human trichuriasis is a whipworm infection caused by the parasitic nematode *Trichuris trichiura*. Approximately 429–508 million people are estimated to be infected with whipworm, with the disease being particularly prevalent in developing countries (1, 2). Current chemotherapeutic interventions involve the administration of albendazole and mebendazole, but these drugs have shown limited efficacy against *T. trichiura* (3–6). As no established animal model exists for human *T. trichiura* infection, most knowledge about trichuriasis has been extrapolated from the well-established laboratory species *T. muris* (mouse whipworm) and *T. suis* (pig whipworm) (7–9). Although many *Trichuris* species exhibit host preferences, recent findings suggest that their host specificity may be more flexible than previously appreciated, with some species (e.g., *T. trichiura* and *T. incognita*) infecting both humans and nonhuman primates or being closely related to animal-infecting taxa (10, 11). Nonetheless, *Trichuris* species share a common tropism for the large intestine, particularly the cecum and proximal colon, within their definitive hosts (7, 12).

Previous studies using *in vivo* mouse models have revealed that *T. muris* infections can lead to either parasite expulsion (acute infection) or persistent infection (chronic infection), depending on factors such as the genetic backgrounds of the mouse strain and the infective dose of *T. muris* eggs. C57BL/6J (B6) mice and STAT6 (signal transducer and activator of transcription 6) knockout (STAT6KO) mice, derived from closely related C57BL/6 substrains, respond differently to high-dose *T. muris* infection due to differences in Th2 immunity. For instance, B6 mice administered a high dose of *T. muris* eggs (*n* = 300) typically clear the infection within 14–21 days, mediated by Th2 immune responses (13, 14). In contrast, high-dose infection in STAT6KO mice results in chronic infection due to impaired Th2 responses (15). Th2 responses are crucial for the expulsion of parasitic nematodes, as they promote mechanisms, such as increased mucus production, epithelial cell turnover, muscle hypercontractility, eosinophil recruitment, and the secretion of cytokines such as interleukin (IL)-4 and IL-13. These effector pathways are regulated in part by STAT6, a key transcription factor in IL-4/IL-13 signaling that is essential for Th2 cell differentiation and immune responses. Its absence disrupts Th2-mediated immune responses, underscoring the importance of STAT6-dependent pathways in the expulsion of parasitic nematodes. Furthermore, genetic variants of STAT6 in humans have been associated with susceptibility to gastrointestinal parasites, highlighting its broader relevance (16).

While B6 and STAT6KO mice represent distinct models of resistance and susceptibility to whipworm infection, the molecular mechanisms underlying these differences still remain unclear. Investigating the early phase of whipworm infection is further complicated by the inability of standard cell lines to support *in vitro* whipworm infection and technical challenges in tracking the first-stage larvae (L1) *in vivo*. L1 larvae preferentially reside at the base of the intestinal crypts, infecting less than 1% of intestinal epithelial cells (IECs), making them difficult to study (17). Understanding how these larvae interact with host environment is critical for unraveling the molecular mechanisms underlying early host–parasite interactions and identifying novel targets for drug interventions before infections progress to chronic stages.

Organoids derived from crypt-residing stem cells—such as those from the cecum or from the proximal colon, regions where whipworms preferentially reside—can proliferate and differentiate *in vitro* into various epithelial lineages, including proliferating cells, goblet cells, and enterocyte and enteroendocrine cells (18). These organoids could potentially provide physiologically relevant components to mimic *in vivo* host–parasite interaction. For example, Eichenberger et al. demonstrated that murine colonic organoids (colonoids) could internalize exosome-like extracellular vesicles (EVs) secreted from adult *T. muris*, suggesting functional host–parasite communication within organoid cultures (19). More recently, mouse cecal organoids (cecaloids), derived from the cecum, another main habitat of whipworm, have been shown to recapitulate key morphological features of early *in vivo* whipworm infection (17). For example, Duque-Correa et al. demonstrated that *T. muris* L1 larvae form syncytial tunnels by burrowing through multiple intestinal epithelial cells (IECs) in cecaloids, closely mimicking *in vivo* infection structures (17). Additional work using cecaloids has enabled investigations of parasite extracellular vesicles and their immunomodulatory functions (20), as well as studies using two-dimensional gastrointestinal monolayers with apical and basal access to examine spatially restricted interactions with helminth-secreted products (21, 22).

Despite these advancements, early transcriptomic profiles between *in vivo* mouse models with different genetic backgrounds (B6 and STAT6KO) and *in vitro* organoid models (e.g., colonoids) have not been compared before. To address this gap, we examined host transcriptional responses during early infection using *in vivo* and *in vitro* (colonoids) models, identifying molecular pathways and genes that were both commonly and differentially induced by L1 *T. muris* infection. In addition, little is known about how whipworm infection influences alternative splicing (AS) in the host intestinal epithelium. AS is a post-transcriptional regulatory mechanism by which multiple transcript isoforms and ultimately diverse protein products can be generated from a single gene (23). This process is evolutionarily conserved across eukaryotes, including mammals, fungi, and parasitic organisms, all of which rely on spliceosomal machinery to regulate essential biological functions (24). In contrast, bacteria and viruses lack intrinsic splicing systems and instead hijack the host’s splicing machinery to modulate gene expression for their survival (24, 25). AS contributes to tissue-specific gene expression and plays roles in developmental programs, the cell cycle, and immune responses (25–28). By examining AS events during L1 *T. muris* infection, we aimed to explore potential splicing-associated regulatory responses that may contribute to the establishment or resolution of infection, independent of gene-level differential expression. Furthermore, we demonstrated *in vitro* whipworm infection in mouse colonoids, suggesting their utility for studying host–parasite interactions. *In vivo* and *in vitro* models are not necessarily expected to be transcriptionally synchronized or identical, as they represent fundamentally different biological contexts. Another potential application of the colonoid model is to evaluate whether it can support early parasite development in a manner resembling its *in vivo* counterparts. In this context, profiling L1 transcriptional dynamics *in vitro* provides a useful benchmark for assessing the suitability of colonoids as a model for early infection. Utilizing dual-RNA sequencing analysis on infected colonoid samples, we broadly captured the transcriptional dynamics of L1 *T. muris* over the course of infection and identified simultaneously expressed host–parasite gene pairs potentially implicated in host–parasite interactions. Our findings contribute to a better understanding of the molecular mechanisms underlying early whipworm infection and provide additional resources to assess the extent to which colonoids, as *in vitro* culture systems of IECs, recapitulate *in vivo T. muris* infection during the early stages.

## RESULTS

### Global comparison of host transcriptomic changes in *in vivo* and *in vitro* models during early *T. muris* infection

**Fig 1:**
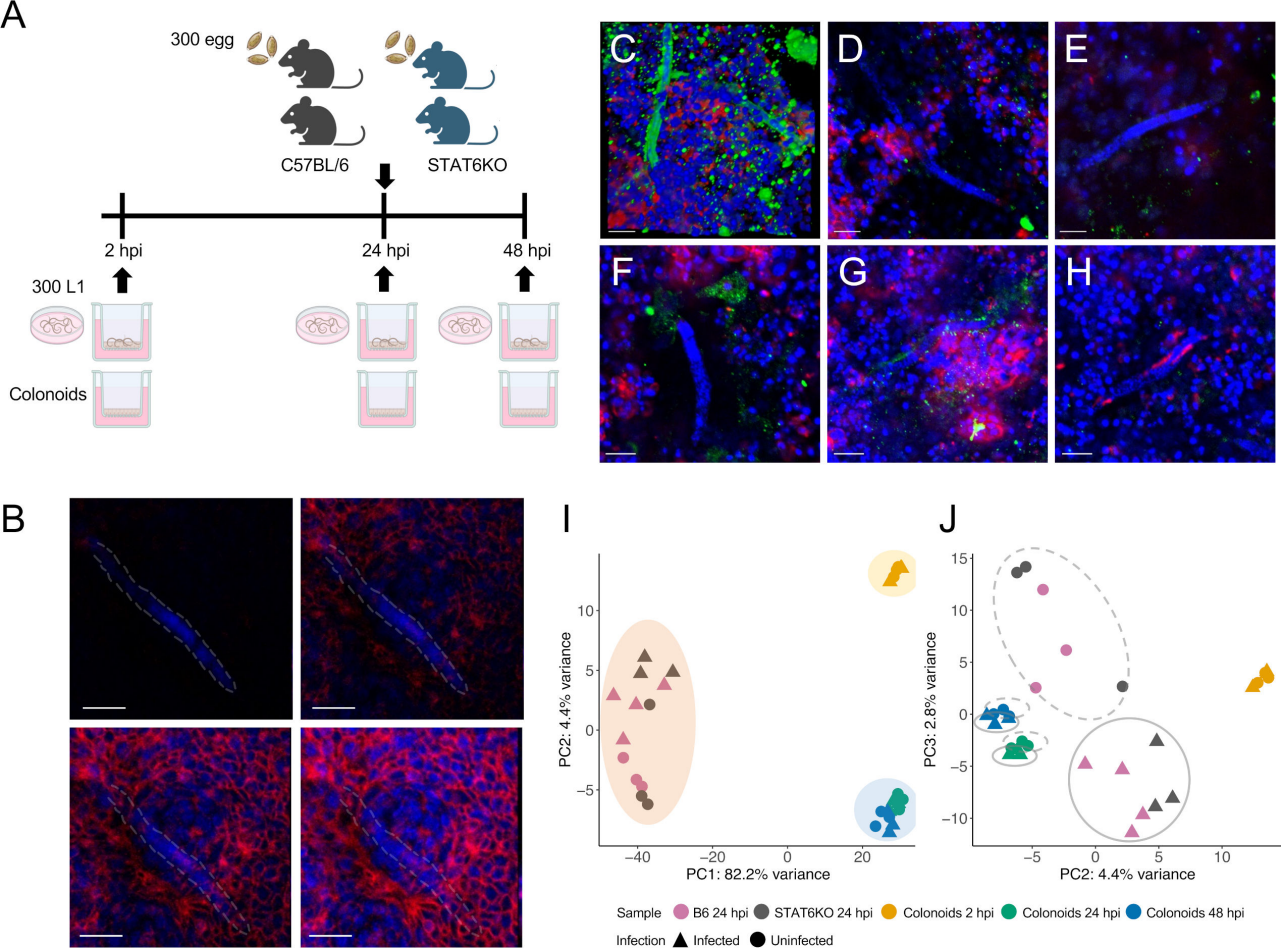
Overview of experimental design and transcriptomic analysis. (**A**) Schematic diagram of *in vivo* and *in vitro* experiments. Top panel: B6 and STAT6KO mice were inoculated with a high (300) dose of infective *T. muris* eggs. Proximal colons were collected at 24 hours post-inoculation (hpi) from infected and uninfected mice. Lower panel: approximately 300 L1 *T*. *muris* larvae were introduced to the apical surface of proximal colonic epithelial monolayer that was differentiated in the air-liquid interface (ALI) for 21 days. The infections were maintained for two, 24, and 48 hours. Uninfected samples were prepared in parallel. For each condition, three to four biological replicates were prepared. (**B–H**) Confocal immunofluorescence (IF) images of proximal colonoids with L1 *T. muris* infection at two hpi (several focal planes shown) (**B**), 24 hpi (**C–E**), and 48 hpi (**F–H**). The monolayer culture was stained with DAPI (nuclei of mouse IECs and *T. muris* L1 larvae, blue), Ki-67 (proliferating cells, green), and phalloidin (F-actin, red). In panel **B**, L1 *T. muris* larva was observed spanning different focal planes. Some parts of the worm appeared clear in one focal plane but faint in another, and vice versa. Dashed white outlines were added to highlight the worm’s morphology across focal planes. Scale bars 20 µm. (**I**) Principal component analysis (PCA) of mouse (host) transcriptomes from *in vivo* and *in vitro* samples, showing PC1 and PC2. (**J**) Principal component analysis (PCA) of mouse (host) transcriptomes from *in vivo* and *in vitro* samples, showing PC2 and PC3.

To investigate *in vivo* transcriptomic responses during the early phase of whipworm infection, we administered a high dose of infective *T. muris* eggs (300 eggs) to mice with two different genetic backgrounds: wild-type B6 and STAT6KO mice (Fig. 1A). These strains were chosen to explore how early transcriptional responses, potentially acting as predictors or molecular alarmins, are modulated in the presence or absence of Th2 signaling. B6 mice possess intact immune, epithelial, neural, and microbial systems and mount a canonical Th2 response, whereas STAT6KO mice retain all these components except functional Th2 signaling, due to disruption of IL-4/IL-13-mediated STAT6 pathways. Proximal colon tissues, one of the primary sites where *T. muris* reside, were collected from both infected and uninfected mice at 24 hours post-inoculation (24 hpi). Experimental infection with our laboratory strain of *T. muris* shows similar levels of parasite burden in the cecum and proximal colon in STAT6KO mice and in B6 mice infected with low doses. Recovery of larval stages in the cecum and proximal colon is also similar in both strains of mice with different doses of *T. muris* eggs, highlighting the equal importance of both intestinal regions for whipworm infection. This time point was selected because it represents a key early stage in the *T. muris* life cycle, when L1 larvae have penetrated the intestinal epithelium and begun forming syncytial tunnels, a hallmark of early host invasion (17). Capturing this stage enables the characterization of initial transcriptional responses associated with larval establishment and early host–parasite interactions. For *in vitro* studies, colonoids were derived from crypts isolated from the proximal colon of B6 mice and differentiated into intestinal epithelial cell (IEC) types, including tuft cells, enteroendocrine cells, and goblet cells as previously described (18) (Fig. S1). Approximately 300 *in vitro*-hatched L1 *T. muris* were introduced to the apical surface of differentiated colonoid monolayers (Fig. 1A). Consistent with previous findings (17), worms appeared to invade deeper into the IEC layer, transitioning to an intracellular position, as demonstrated by their presence in different focal planes within the colonoids at two hpi (Fig. 1B). Over time, L1 *T. muris* were frequently observed near Ki-67+ proliferating IECs during the early stages of infection, from 24 hpi through 48 hpi (Fig. 1C through H). The co-localization of green Ki-67 signals with the surface was observed for some, but not all, worms, indicating that these worms were adjacent to Ki-67 + proliferating ICEs as previously observed in cecaloids during whipworm infection (17). We also observed that the colonoid model could support *T. muris* infection beyond the early stages (e.g., 48 hpi). Larvae persisted in the monolayer culture for up to 14 days post-infection (dpi), forming syncytial tunnels and exhibiting signs of growth, including increased body size and structural changes (Fig. S2). Preliminary measurements indicated that worm length increased from approximately 100  µm at early stages (2–48 hpi) to a range of 120–350  µm by 14 dpi, with widths increasing from ~7 µm to ~17 µm (Fig. S2G and H). One worm observed at 14 dpi (Fig. S2D) remained relatively small (~ 120  µm in length), possibly reflecting a slower-growing larva or one that hatched later. Additionally, anterior DAPI-stained structures, likely corresponding to the nerve ring, were visible in L1 larvae at early time points but were less apparent at later stages, suggesting potential morphological changes. While these observations are based on limited sample numbers and should be interpreted with caution, they support the potential of the colonoid model to sustain whipworm development beyond the initial stages of infection.

To examine *in vitro* transcriptional responses during the early stages of the infection, transcriptional profiles of colonoids at two, 24, and 48 hpi were compared with transcriptional profiles of uninfected colonoids, generated using bulk RNA-seq based approach. Principal component analysis (PCA) of transcriptional expression profiles revealed distinct segregation of samples along the first principal component (PC1), which accounted for 82.2% of the variance and distinguished *in vivo* mouse samples from *in vitro* colonoid samples (Fig. 1I). The second principal component (PC2), explaining 4.4% of the variance, separated two-hour colonoid samples from the 24- and 48-hour colonoid samples, indicating temporal changes in transcriptional profiles within the colonoid model. PC3, although accounting for a smaller proportion of the variance (approximately 2.8%), enhanced the resolution of separation between infected and uninfected colonoid samples at the 24- and 48-hour time points compared to the tw0-hour time point (Fig. 1J). For *in vivo* samples, PC2 and PC3 captured the overall infection status across genetic backgrounds (B6 and STAT6KO), as infected and uninfected samples were distinctly separated along these components.

Differential gene expression analysis was performed between uninfected and infected samples within each cohort, using DESeq2 (29) (Tables S1 and S2). In mice, samples collected at 24 hpi identified 239 significantly upregulated and 295 significantly downregulated genes in the infected B6 mice compared to the uninfected mice at 24 hpi, while 96 significantly upregulated and 272 significantly downregulated genes were identified in STAT6KO (Table 1). In colonoids, the number of significantly differentially expressed genes (DEGs) between infected and uninfected samples decreased over time, from 587 at two hpi to 99 at 24 hpi and 80 genes at 48 hpi. An analysis of alternative splicing (AS) events revealed the highest number of AS events (537) in STAT6KO mice at 24 hpi (Table 2). AS events in colonoids exhibited dynamic changes throughout the infection progression (two, 24, and 48 hpi), contrasting with the observed decrease in the number of DEGs. Exon skipping was the most frequent AS event identified in both *in vivo* mice models and *in vitro* colonoids. These observations indicate dynamic changes in host transcriptional responses during early whipworm infection in both *in vivo* mice and *in vitro* colonoid model system.

**TABLE 1:** A summary of differentially expressed genes (DEGs) analysis between uninfected and infected samples of each experiment with fold change ≥ 1.3 and FDR-adjusted *P*-value (P_adj_) ≤ 0.05 for significance

Differentially expressed genes	Infected vs. uninfected gene counts					
	*In vivo*, mice, 24 hpi			*In vitro*, colonoids		
	B6	STAT6KO^*a*^	Overlap	2 hpi	24 hpi	48 hpi
Up	239	96	10	282	15	26
Down	295	272	86	305	84	54
Total	534	368	96	587	99	80

^*a*^ Knockout mice.

**TABLE 2:** A summary of identified alternative (AS) exons by alternative splicing type in each condition with differential percent spliced in (PSI) ≥ 0.05 and FDR-adjusted *P*-value (*P*_adj_) ≤ 0.1 for significance

Differential alternative splicing event^*b*^	Infected vs. uninfected gene counts					
	*In vivo*, mice, 24 hpi			*In vitro*, colonoids		
	B6	STAT6KO^*a*^	Overlap	2 hpi	24 hpi	48 hpi
ES	108	274	6	180	110	97
IR	83	164	8	91	87	70
A3SS	33	101	2	63	59	41
A5SS	21	69	1	23	24	33
MXE	11	30	2	30	7	9
Total	214	537	48	329	267	228

^*a*^ Knockout mice.

^*b*^ ES, exon skipping; IR, intron retention; A3SS, alternative 3' splicing site; A5SS, alternative 5' splicing site; MXE, mutually exclusive exons.

### Characterization of transcriptional responses of colon tissue from B6 mice during early infection with *T. muris*

**Fig 2:**
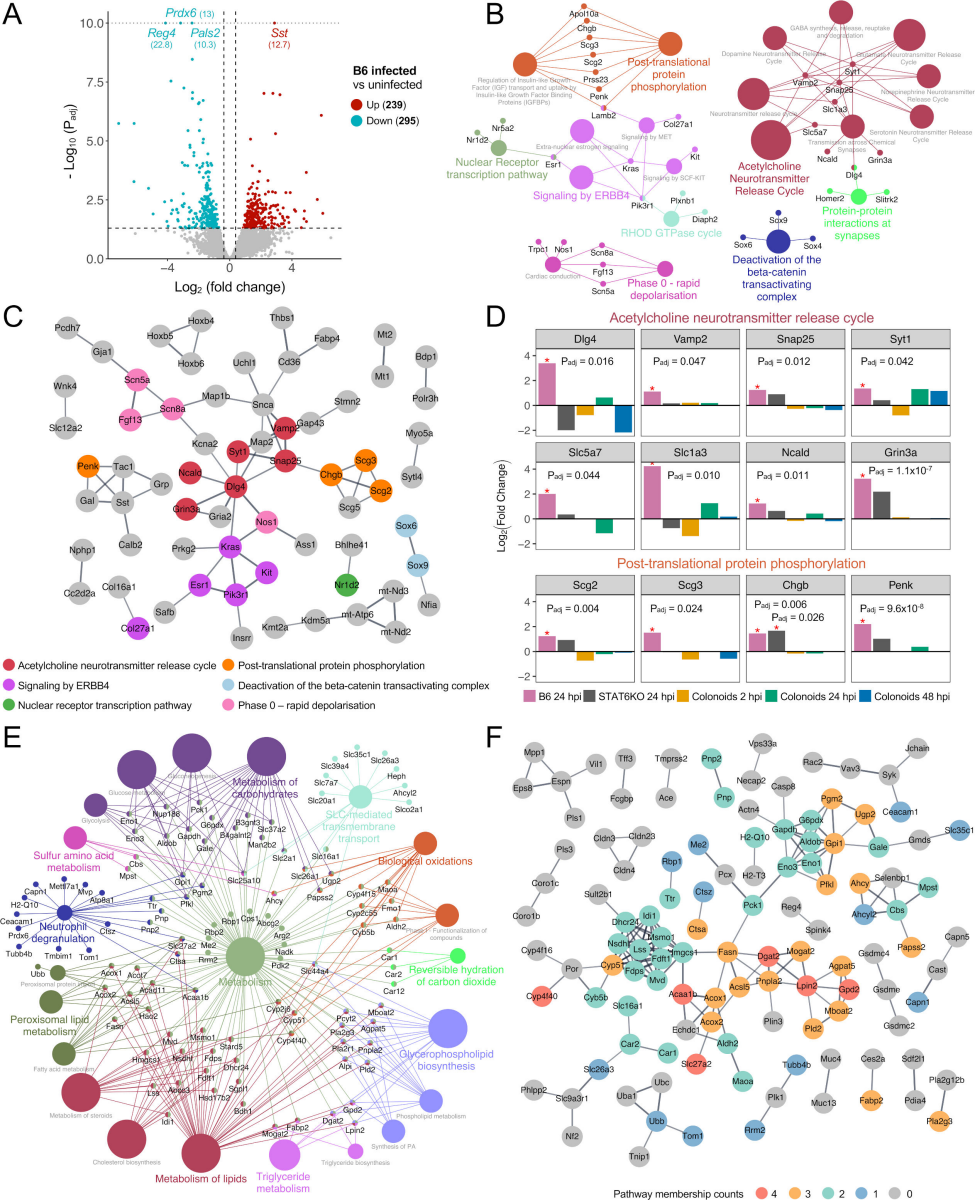
Host transcriptional response in infected B6 mice during the early infection with L1 *T. muris*. (**A**) Volcano plot generated using DEGs in infected B6 mice at 24 hpi compared to uninfected B6 mice, with fold change ≥ 1.3 and *P*_adj_ ≤ 0.05 for significance. Some of the DEGs were selectively labeled based on their statistical significance and biological relevance to the pathways highlighted in Results. For clarity and improved visualization, *y*-axis scaling was adjusted by aligning highly significant DEGs with the maximum -log_10_(*P*_adj_) line to avoid excessively long axis ranges. (**B, E**) Significantly enriched Reactome pathways were identified using the ConsensusPathDB and visualized in network diagrams using the ClueuGo/CluePedia plugin in Cytoscape. (**C, F**) Protein-protein interaction (PPI) network of DEGs was identified between infected B6 mice at 24 hpi and uninfected control using the STRING database. Each node represents one protein, and edges between nodes represent interactions. Nodes were colored by either significantly enriched pathways (**C**) or pathway membership counts (**F**). Disconnected nodes were removed, and minimum interaction confidence was set to 0.700. (**D**) Bar plot representing log_2_ fold change of mouse genes after inoculation in B6 (24 hpi) and STAT6 KO (24 hpi) mice and in mouse proximal colonoids (2, 24, and 48 hpi), involved in the acetylcholine neurotransmitter release cycle or post-translational protein phosphorylation pathways. Statistical significance (*P*_adj_) in differential expression between infected and uninfected mice is highlighted with a red asterisk. Panels **B** and **C** correspond to significantly upregulated genes in infected B6 mice at 24 hpi compared to control. Panels **E** and **F** correspond to significantly downregulated genes in infected B6 mice at 24 hpi compared to control.

In response to *T. muris* infection, 239 and 295 genes were significantly up- and downregulated in the infected B6 mice compared to the uninfected mice at 24 hpi (Fig. 2A; Table S2). *Sst*, encoding a peptide hormone called somatostatin, was the most significantly upregulated gene (*P*_adj_ = 2.3 × 10^−13^ and log_2_ fold change = 2.9). Functional enrichment analysis of the upregulated genes identified significantly enriched functional pathways, including acetylcholine neurotransmitter release cycle (*P*_adj_ = 3.0 × 10^−3^), neuronal system (5.8 × 10^−3^), post-translational protein phosphorylation (5.8 × 10^−3^), deactivation of the beta-catenin transactivating complex (3.3 × 10^−2^), and signaling by ERBB4 pathways (3.9 × 10^−2^) (Fig. 2B; Tables S3 and S4).

Protein-protein interaction (PPI) network analysis using STRING (30) identified 211 nodes and 73 edges in the connected upregulated gene network at a high confidence interaction score of 0.7, indicating significantly more interactions than expected by random chance (*P* = 1.6 × 10^−15^; Fig. 2C; Table S5). The top five hub genes with the highest node degrees were *Dlg4*, *Kras*, *Snca*, *Snap25*, and *Sst*, of which *Dlg4* and *Snap25* were associated with acetylcholine neurotransmitter release cycle pathway (Fig. 2B; Table S6). Additional genes involved in this pathway, such as *Vamp2*, *Syt1*, *Slc5a7*, *Slc1a3*, *Ncald*, and *Grin3a*, were significantly upregulated in B6 mice after infection (Fig. 2D). Among these, *Snap25* was identified as a hub gene interacting with chromogranin (*Chgb*) (Fig. 2C). *Chgb*, along with other hormones and peptide precursors, such as secretogranins (*Scg2* and *Scg3*) and proenkephalin (*Penk*), was associated with secretory vesicles and enriched in the post-translational protein phosphorylation pathway (Fig. 2B). These genes were predominantly upregulated in B6 mice, but not in STAT6KO mice or murine colonoids, during the early *T. muris* infection (Fig. 2D).

Of the 295 downregulated genes in *T. muris*-infected B6 mice at 24 hpi, *Reg4* (*P*_adj_ = 1.7 × 10^−23^ and log_2_ fold change = −4.1), a marker for deep secretory cells (DSCs) at the crypt base, was the most significantly downregulated gene (Fig. 2A). DSCs were identified as one of the main constituents of the syncytial tunnel hosting L1 *T. muris* in the cecum of mice at 24 hpi (17). *Prdx6* (*P*_adj_ = 1.1 × 10^−13^ and log_2_ fold change = −3.2), the second-most significantly downregulated gene, is involved in redox regulation of cells and plays a role in the regulation of glucose homeostasis, lipid metabolism, inflammation, and cell proliferation (31, 32).

Functional enrichment analysis of the downregulated genes identified 36 significantly enriched pathways, with the top five being related to metabolism: metabolism (*P*_adj_ = 3.2 × 10^−23^), metabolism of lipids (4.3 × 10^−13^), cholesterol biosynthesis (4.6 × 10^−10^), gluconeogenesis (2.3 × 10^−8^), and metabolism of carbohydrate (3.2 × 10^−8^) (Fig. 2E; Tables S7 and S8). The PPI network of the downregulated genes comprises 279 nodes and 166 edges (*P* < 1.0 × 10^−16^; Fig. 2F; Table S9). The top five genes (*Cyp51*, *Hmgcs1*, *Dhcr24*, *Fdft1*, and *Fdps*), all associated with the metabolism of lipids pathway, highlighted transcriptional modulation of lipid-related processes (Table S10). Additionally, some genes (*Dgat2*, *Lpin2*, and *Gpd2* highlighted with orange in Fig. 2F) were involved in four different metabolism pathways, suggesting broad transcriptional changes in metabolism processes during infection.

### Characterization of transcriptional responses of colon tissue from STAT6KO mice and comparison with B6 mice during early *T. muris* infection

**Fig 3:**
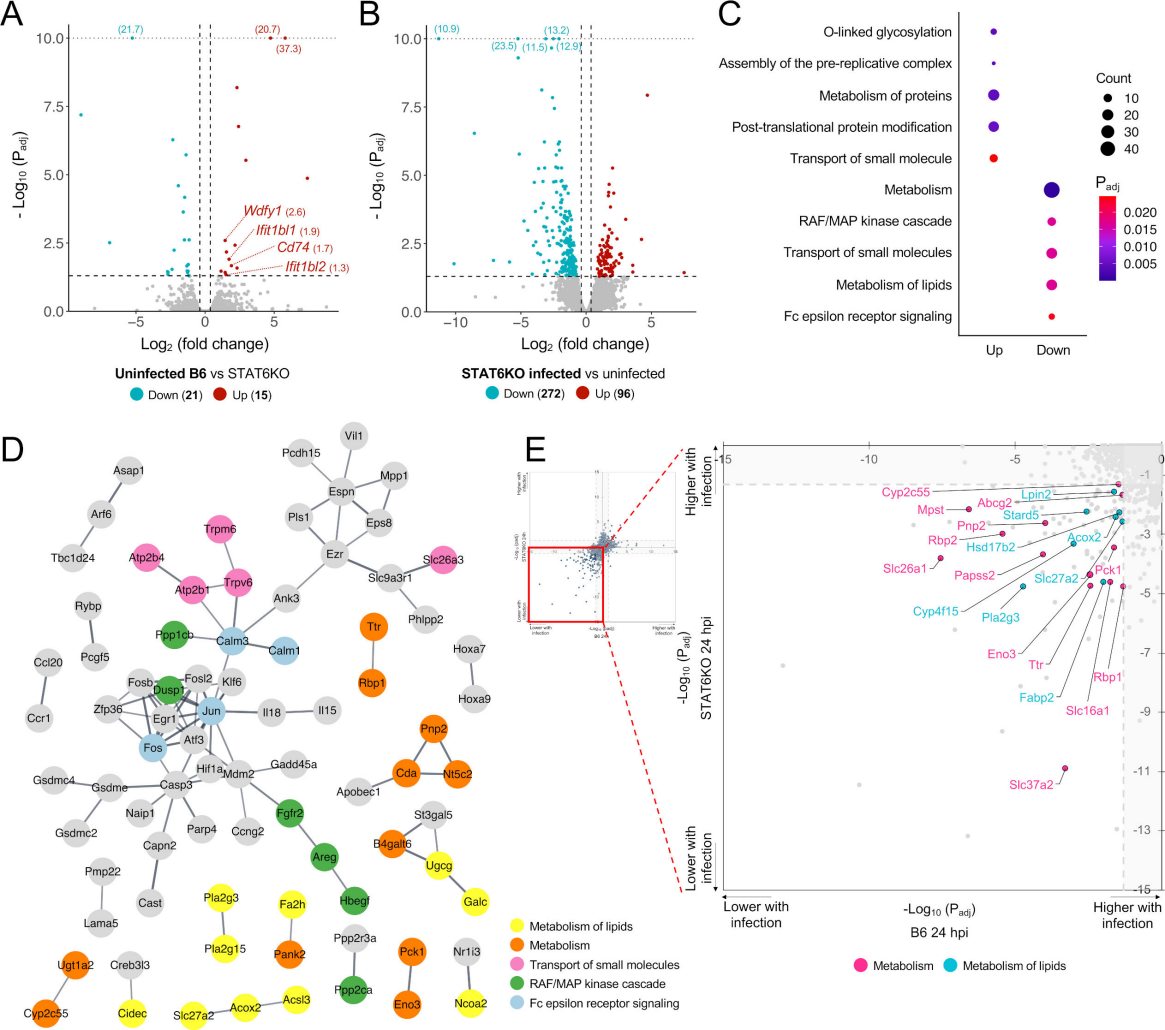
Host transcriptional response in infected STAT6KO mice during the early infection with L1 *T. muris*. (**A**) Volcano plot generated using DEGs in uninfected B6 mice compared to uninfected STAT6KO mice, with fold change ≥ 1.3 and *P*_adj_ ≤ 0.05 for significance. Some of the DEGs were selectively labeled based on their statistical significance and biological relevance to the pathways highlighted in Results. For clarity and improved visualization, *y*-axis scaling was adjusted by aligning highly significant DEGs with the maximum -log_10_(*P*_adj_) line to avoid excessively long axis ranges. (**B**) Volcano plot generated using DEGs in infected STAT6KO mice compared to uninfected STAT6KO mice at 24 hpi, with fold change ≥ 1.3 and *P*_adj_ ≤ 0.05 for significance. (**C**) Significantly enriched Reactome pathways associated with significantly up- and downregulated genes in infected STAT6KO mice at 24 hpi. The *P*_adj_ (*q*-value) for each pathway is represented by the color, and the number of significant DEGs from each pathway is represented by the dot size. (**D**) Protein-protein interaction (PPI) network of significantly downregulated genes identified in infected STAT6KO mice at 24 hpi using the STRING database. Each node represents one protein, and edges between nodes represent interactions. Nodes were colored by significantly enriched pathways. Disconnected nodes were removed, and minimum interaction confidence was set to 0.700. (**E**) Scatter plot of -log_10_(*P*_adj_) of DEGs in and B6 mice at 24 hpi and STAT6KO mice at 24 hpi. Significantly upregulated genes were represented as positive log_10_(*P*_adj_) values, while significantly downregulated genes were represented as negative log_10_(*P*_adj_) values. The red inset highlights a part of common DEGs in both infected B6 mice and infected STAT6KO mice. Genes represented by orange dots are involved in the metabolism pathway, and genes represented by yellow dots are involved in the metabolism of lipids.

In the absence of *T. muris* infection, only 36 genes (15 upregulated and 21 downregulated genes) showed differential expression between B6 and STAT6KO mice (Fig. 3A; Table S2). Of the 15 upregulated genes, four (*Ifit1bl1*, *Ifit1bl2*, *Wdfy1*, and *CD74*) were associated with innate immune responses. However, none of the DEGs, in the absence of infection, appeared to be directly linked to adaptive immune responses, such as Th1/Th2 cytokine responses, which are crucial for determining protection against the *T. muris* infection. These findings indicate minimal differences in immune-related gene expression profiles between B6 and STAT6KO mice in the absence of *T. muris* infection.

In *T. muris*-infected STAT6KO mice, 96 and 272 genes were significantly up- and downregulated, respectively, compared to uninfected STAT6KO control (Fig. 3B). The transcriptional changes induced by *T. muris* infection, especially during the early phase*,* were more pronounced than those observed between uninfected B6 and STAT6KO mice (Fig. 3A and B).

Functional enrichment analysis with the 96 upregulated genes identified significantly enriched pathways, including O-linked glycosylation, assembly of the pre-replicative complex, metabolism of protein, post-translational protein modification, and transport of small molecule (Fig. 3C; Table S11). The PPI network for upregulated genes was composed of nodes (*n* = 95) and edges (*n* = 14) (Tables S12 and S13).

Among the 272 downregulated genes, enriched pathways included an immune response pathway (*P*_adj_ = 2.4 × 10^−5^), the Fc epsilon receptor signaling pathway (Fig. 3C; Table S14). In a PPI network consisting of 245 nodes and 95 edges (more than expected by random chance, *P* < 1.0 × 10^−16^), the transcription factor *Jun* and protein *c-Fos*, forming a transcriptional regulator known as activator protein 1 (AP-1) (33), appeared as top hub genes with the highest node degrees in the network (Fig. 3D; Tables S15 and S16). *Calm1* and *Calm3* were associated with the Fc epsilon receptor signaling pathway, which regulates the production of cytokines, such as IL-3, IL-4, IL-5, and IL-13. Furthermore, two cytokine genes (*Il15* and *Il18*) were significantly downregulated in the infected STAT6KO mice at 24 hpi (Table S2). Pathways, such as metabolism (*P*_adj_ = 5.0×10^−6^), RAF/MAP kinase cascade (1.7 × 10^−2^), transport of small molecule (1.7 × 10^−2^), and metabolism of lipid (1.7 × 10^−2^), were also enriched among the downregulated genes in STAT6KO mice (Fig. 3C). The transport of small molecule pathway was observed in both upregulated and downregulated gene sets. Pathways related to metabolism and metabolism of lipid were commonly identified as downregulated in both *T. muris*-infected B6 and STAT6KO mice at 24 hpi (Fig. 2E and 3C).

A comparison of DEGs between *T. muris*-infected STAT6KO and B6 mice at 24 hpi identified 10 commonly upregulated and 86 commonly downregulated genes (Table 1). Among the commonly downregulated genes, 21 were predominantly associated with the metabolism pathway, and eight genes (*Lpin2*, *Stard5*, *Hsd17b2*, *Acox2*, *Slc27a2*, *Cyp4f15*, *Fabp2*, and *Pla2g3*, highlighted as cyan dots in Fig. 3E) were involved in the metabolism of lipid pathway. These genes are all linked to lipid homeostasis and related physiological processes of the mouse intestine. Taken together, the common molecular features suggested the shared transcriptional modulations of host metabolism pathways in the proximal colon of both B6 and STAT6KO mice during L1 *T. muris* infection.

### Host transcriptional responses in proximal colonoids derived from B6 mice and comparison to colon from B6 and STAT6KO mice during early *T. muris* infection

**Fig 4:**
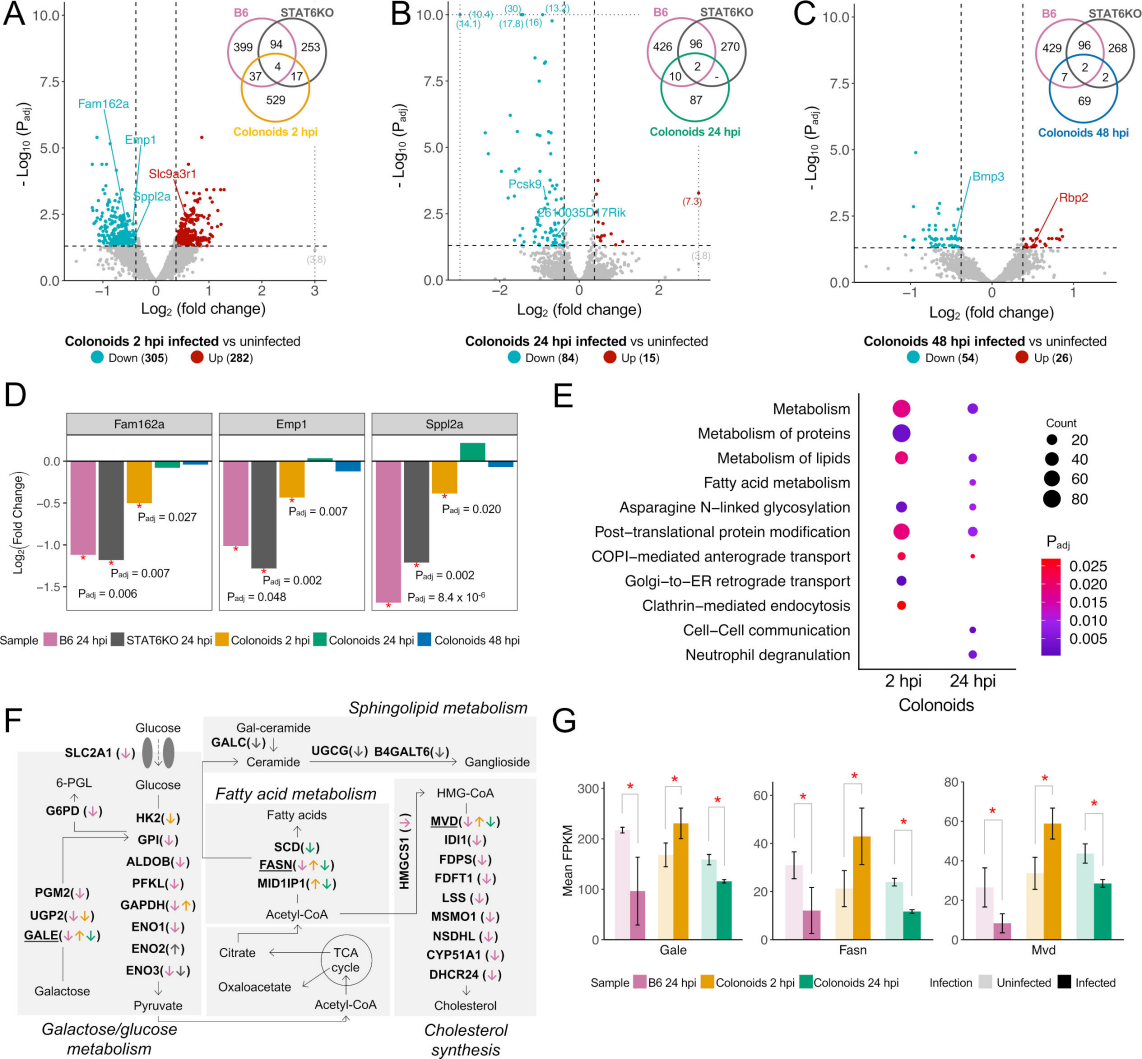
Host transcriptional response in infected proximal colonoids during the early infection with L1 *T. muris*. (**A, B, C**) Volcano plots generated using DEGs in infected colonoids compared to uninfected controls at corresponding infection time points (2, 24, and 48 hpi), with fold change ≥ 1.3 and *P*_adj_ ≤ 0.05 for significance. Some of the DEGs were selectively labeled based on their statistical significance and biological relevance to the pathways highlighted in Results. For clarity and improved visualization, *y*-axis scaling was adjusted by aligning highly significant DEGs with the maximum -log_10_(*P*_adj_) line to avoid excessively long axis ranges. A three-way Venn diagram of common DEGs from B6 mice at 24 hpi, STAT6KO mice at 24 hpi, and colonoids at two, 24, and 48 hpi. (**D**) Bar plot representing log_2_ fold change of three mouse genes (*Fam163a*, *Emp1*, and *Sppl2a*) commonly downregulated after infection in B6 mice (24 hpi), STAT6KO mice (24 hpi), and mouse proximal colonoids (two hpi). Statistical significance (*P*_adj_) in differential expression between infected and uninfected mice is highlighted with a red asterisk. (**E**) Significantly enriched Reactome pathways associated with DEGs in colonoids at two and 24 hpi. The *P*_adj_ (*q*-value) for each pathway is represented by color, and the number of significant DEGs from each pathway is represented by dot size. (**F**) Simplified metabolism pathways including galactose, glucose, fatty acid, sphingolipid metabolism, and cholesterol synthesis pathways. Significant DEGs identified in B6 mice at 24 hpi, STAT6KO mice at 24 hpi, and colonoids at two, 24, and 48 hpi are shown with corresponding group colors. Upright and downward arrows represent significantly up- and downregulated genes, respectively. (**G**) Box plot representing the average of normalized expression values (FPKM) of three representative genes (*Gale*, *Fasn*, and *Mvd*) highlighted in panel **E** for infected and uninfected samples from B6 mice at 24 hpi and colonoids at two and 24 hpi.

A recent study has demonstrated the use of cecaloids to study the early *T. muris* infection *in vitro* (17), but a transcriptomic landscape over the course of the infection, particularly in the proximal colon and *in vitro* colonoid systems, has yet to be fully explored. To complement and expand on the previous study, we characterized host transcriptional profiles during L1 *T. muris* infection in *in vitro* colonoids by performing a comparative analysis of infected versus uninfected samples across three distinct time points (Fig. 4A through C). The number of DEGs showed a decreasing trend in as infection progressed in colonoids: 587 DEGs (282 upregulated and 305 downregulated) at two hpi, 99 DEGs (15 upregulated and 84 downregulated) at 24 hpi, and 80 DEGs (26 upregulated and 54 downregulated) at 48 hpi (Table 1). Of note, 36 DEGs (11 upregulated and 25 downregulated) were shared between two and 48 hpi colonoid samples (Table S2).

Comparison of DEGs among colonoids and infected B6 and STAT6KO mice at 24 hpi revealed minimal overlap: four genes (*Fam162a*, *Slc9a3r1*, *Sppl2a*, and *Emp1*) at two hpi, two genes (*Pcsk9* and 2610035D17Rik) at 24 hpi, and two genes (*Bmp3* and *Rbp2*) at 48 hpi (Fig. 4A through C). Among these, only three genes (*Fam162a*, *Emp1*, and *Sppl2a*) showed consistent downregulation in both colonoids at two hpi and *in vivo* models at 24 hpi (Fig. 4D).

Functional enrichment analysis of DEGs in colonoids identified pathways shared between two and 24 hpi, but not at 48 hpi, including metabolism, metabolism of lipids, asparagine N-linked glycosylation, post-translational protein modification, and COPI-mediated anterograde transport (Fig. 4E; Tables S17 and S18). Comparative analysis highlighted transcriptional changes in metabolism pathways related to galactose, glucose, lipid, sphingolipid, and cholesterol metabolisms across *in vitro* and *in vivo* models (Fig. 4F). For example, *Gale*, *Fasn,* and *Mvd* genes regulating galactose metabolism, fatty acid metabolism, and cholesterol synthesis, respectively (34–36), were significantly downregulated in *T. muris*-infected B6 mice at 24 hpi (Fig. 4G). In infected colonoids, these genes showed an initial upregulation at two hpi, followed by downregulation at 24 hpi, mirroring the downregulation expression patterns observed in *in vivo* infected B6 mice at 24 hpi.

### Alternative splicing analysis of B6 mice, STAT6KO mice, and colonoids during early *T. muris* infection

Considering the regulatory importance of alternative splicing (AS) in gene expression and its potential role in host–parasite interactions, we analyzed differential AS events in B6 mice, STAT6KO mice, and colonoids during early *T. muris* infection (37–39). A total of 214, 537, 329, 267, and 228 differential AS genes were identified between infected and uninfected conditions in B6 mice, STAT6KO mice, and colonoids at two, 24, and 48 hpi, respectively (Table 2; Tables S19 to S23).

Functional enrichment analysis revealed that the apoptosis-related pathway (*P*_adj_ = 7.6 × 10^−2^) was identified only in B6 mice (Fig. S3A; Table S24). Genes associated with this pathway, including *Add3*, *Appl1*, *Rock1*, *Dbnl*, and *Mapt*, exhibited significant differences in exon percent spliced in (PSI) values between infected and uninfected B6 mice (*P*_adj_ ≤0.05) (Fig. S3B). Conversely, the mRNA splicing pathway was significantly enriched in both B6 (*P*_adj_ = 7.6 × 10^−2^) and STAT6KO (*P*_adj_ = 2.7 × 10^−2^) mice, with notable AS events observed in genes such as *Sirt7* and *Srsf5* (Fig. S3C). In both mouse strains, *Sirt7* exhibited a reduction in retained intron transcript forms, whereas *Srsf5* showed increased retained intron forms in infected conditions compared to uninfected controls. *Tia1* showed significant differences in PSI values across B6 mice, STAT6KO mice, and colonoids at 24 hpi (*P*_adj_ ≤ 0.05) and moderate differences in colonoids at two and 48 hpi (*P*_adj_ ≤ 0.1) (Fig. S3D).

To explore common AS features across mouse strains and colonoids, shared AS genes and functional pathways were examined. The mRNA splicing pathway, enriched in both mouse strains, was consistently enriched in colonoids at all time points (Fig. S4A). Shared AS genes were identified across both mouse strains and colonoid samples, with 10, nine, and 10 genes identified in colonoids at two, 24, and 48 hpi, respectively (Fig. S4B). However, most AS events in the splicing pathway were specific to individual samples and did not overlap at the exon level among the samples (e.g., B6, STAT6KO, colonoids at two, 24, and 48 hpi) (Fig. S4C). For example, different exons of *Hnrnph1*, an alternative splicing regulator, were alternatively spliced depending on infection conditions (Fig. S4D). These results indicate that alternative splicing events are dynamically regulated in the host during early *T. muris* infection, with specific pathways, such as mRNA splicing and apoptosis, showing distinct modulation patterns depending on host model and time point.

### Temporal profiling of L1 *T. muris* transcriptome during early infection of colonoids derived from B6 mouse

To investigate the transcriptomic dynamics of L1 *T. muris* during early infection, we utilized dual-RNA sequencing in proximal colonoid samples collected at two, 24, and 48 hpi, in addition to examining host transcriptional responses (Fig. 1A; Table S25).

**Fig 5:**
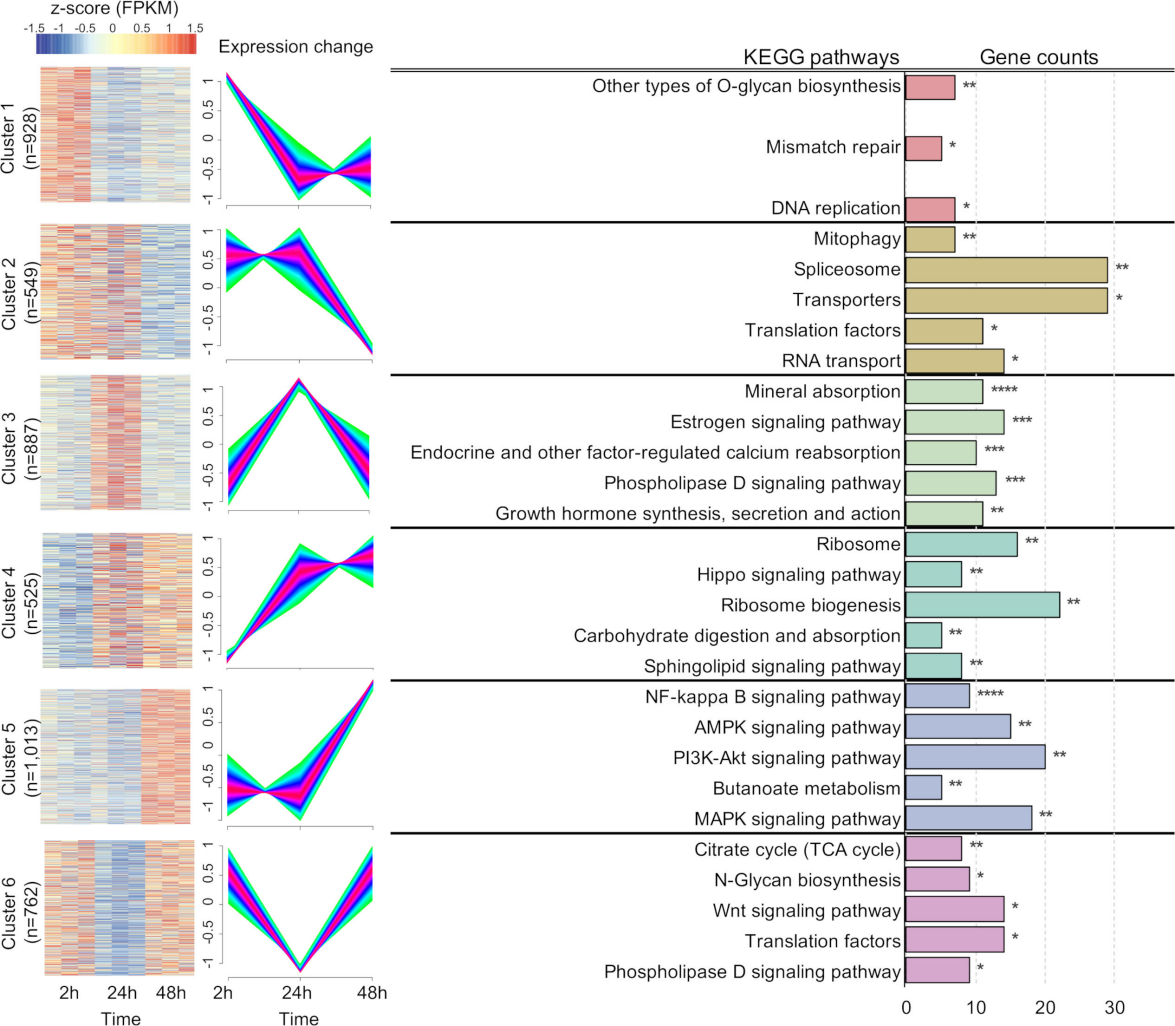
Temporal transcriptional responses of L1 *T. muris* in proximal colonoids. Heatmaps represent the z-scores of FPKM values for *T. muris* genes grouped in each cluster. Columns of the heatmap indicate infection time points for each infected colonoid sample. A total of six clusters were identified using 10 iterations of Mfuzz soft clustering with an FCM parameter (m) of 2.71. A membership value in the range of 0–1 was assigned in clustering, and the cluster cores consisting of genes with membership value ≥0.90 were colored pink. For each cluster, enriched KEGG pathways are shown in the bar plot with *P*-value thresholds for significance indicated as follows: *P ≤ 0.05, **P ≤ 0.01, ***P ≤ 10^−3^, ****P ≤ 10^−4^, *****P ≤ 10^−5^.

As expected with dual-RNA seq, sequencing primarily captured host-derived reads, with *T. muris* reads representing only a small fraction of total mapped reads (approximately 0.4% on average). This low mapping rate was consistent across colonoid infection time points (two, 24, and 48 hpi), prompting us to apply minimal-expression filtering followed by trajectory-based clustering to recover biologically informative parasite gene expression dynamics. From the total 14,995 *T. muris* genes, 5,246 genes with consistent expression across at least two of three biological replicates at any infection time point were retained. To focus on genes exhibiting meaningful expression changes, 4,701 genes with a maximum log_2_ fold change ≥0.5 among three pairwise comparisons (2 vs. 24 hpi, 2 vs. 48 hpi, and 24 vs. 48 hpi) were further analyzed. Of these, 4,664 genes were grouped into six clusters based on their expression trajectories using Mfuzz soft clustering (40), achieving ≥99.46% stability in cluster membership across 10 iterations (Fig. 5; Table S26). Functional enrichment analysis revealed dynamic shifts in biological pathways over the course of infection (Table S27).

Genes in cluster 1, characterized by high expression at two hpi, were enriched in pathways related to mismatch repair and DNA replication factors, as well as O-glycan biosynthesis. Seven genes encoding proteins with glycosyltransferase activities were identified, suggesting a role for glycosylation processes during the early stages of infection.

Cluster 2, with genes upregulated at two and 24 hpi, was associated with pathways such as mitophagy, spliceosome, transporters, translation factors, and RNA transport. Among a total of 315 genes in transporter pathways identified in *T. muris*, 29 genes within cluster 2 included TMUE_3000014900, orthologous to *pmp-3* (peroxisomal membrane protein related) in *C. elegans* and *ABCD4* in human, which is predicted to have fatty acid (lipid) transporter activity (41).

Functional enrichment of genes in cluster 3 (uniquely upregulated at 24 hpi) resulted in identification of transporters for mineral absorption and kinases in several signaling pathways.

Cluster 4, which showed upregulation at 24 and 48 hpi relative to two hpi, represented the lowest number of gene set (*n* = 525) among the six clusters. Two cellular ion transporters, orthologous to *eat-6* and *egl-19* in *C. elegans*, respectively, were involved in carbohydrate digestion and absorption pathways. These two genes play important roles in controlling pharyngeal muscle of *C. elegans* (42, 43). Other enriched pathways included ribosome biogenesis and kinase signaling pathways, suggesting involvement in cell growth, proliferation, and differentiation at later stages of infection.

Cluster 5 displayed the highest number of genes (*n* = 1,013), characterized by elevated expression at 48 hpi, and was enriched in pathways, such as NF-kappa B, AMPK, PI3K-akt, and MAPK signaling. Additionally, five genes related to butanoate metabolism pathway were identified, suggesting increased utilization of short-chain fatty acids for energy metabolism or other metabolic processes at the later stages of infection.

Cluster 6 comprised genes upregulated at both two and 48 hpi, with enrichment in pathways, such as the citrate cycle (TCA cycle) and N-glycan biosynthesis. These findings indicate potential involvement of glycosylation and energy metabolism during these time points.

In conclusion, the temporal profiling of L1 *T. muris* transcriptome resulted in identification of dynamic changes in gene expression and fundamental biological processes that could support cellular activity and fulfill metabolic demands during the early stage of the infection.

### Co-expression analysis between L1 *T. muris* and proximal colonoids during early phase infection

**Fig 6:**
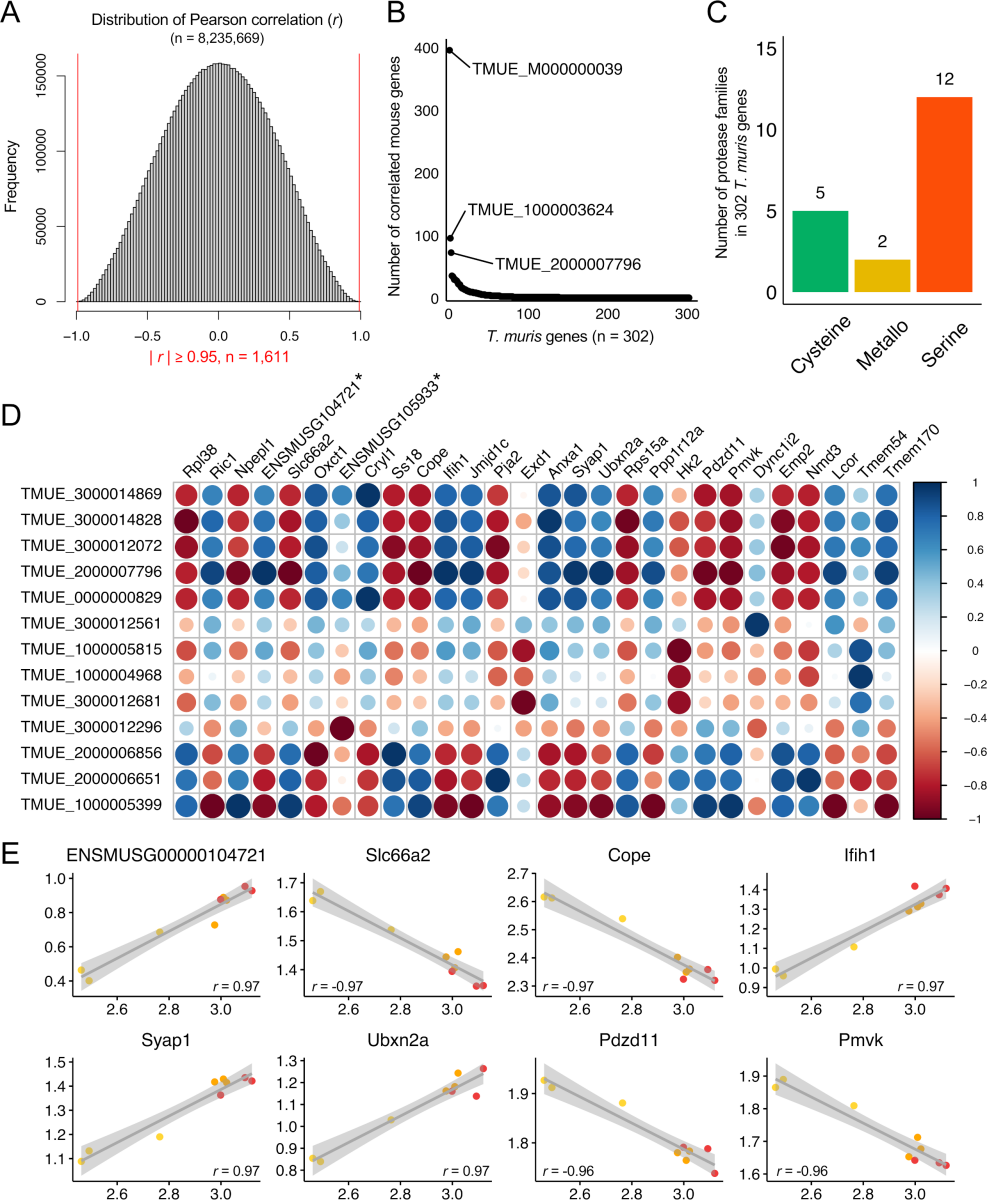
Expression correlation between host (mouse proximal colonoids) and parasite (L1 *T. muris*). (**A**) Distribution of Pearson correlations (*r*) among a total of 8,235,669 gene pairs detected in three biological replicates of L1 *T. muris* and mouse colonoids at all three infection time points (two, 24, and 48 hpi). A total of 1,611 gene pairs showed a strong correlation (| *r* | ≥ 0.95) between *T. muris* and mouse. (**B**) Dot plot illustrating the number of highly correlated mouse genes per *T. muris* gene. (**C**) Identification of protease families within 302 *T. muris* genes that strongly correlated (| *r* | ≥ 0.95) with mouse genes. (**D**) Correlation plot representing the top 30 gene pairs based on Pearson correlation (*r*) values after prioritizing predicted secreted *T. muris* genes and differentially expressed mouse genes between infected and uninfected colonoids at any of the three infection time points, with *P*-value ≤ 0.05 threshold for significance. The original Ensembl gene IDs (e.g., “ENSMUSG00000104721” and “ENSMUSG00000105933”) were abbreviated by removing the zeros to save space in the figure, and this is highlighted with asterisks in the figure. (**E**) Scatter plots showing log_10_ FPKMs of mouse genes (*y*-axis) highly correlated with *T. muris* TMUE_2000007796 gene (*x*-axis) at the three infection time points (two, 24, and 48 hpi). Pearson correlation (*r*) values are indicated for each gene pair.

To investigate potential transcriptional coordination between host and parasite genes during early infection, we performed a gene-level Pearson correlation analysis using expression values from infected colonoids across all three time points. This approach aimed to identify gene pairs whose expression levels were tightly correlated, which may reflect molecular cross-talk or coordinated biological responses during early infection. We first prioritized a subset of *T. muris* and mouse genes expressed in all three biological replicates at all time points, excluding genes with zero-level expression to minimize false-positive correlations (see Methods). Gene pairs with strong correlation (|*r*| ≥ 0.95) were retained. Using this criteria, one gene can be paired with multiple genes from the other species, so the final set represented 1,611 highly correlated *T. muris*-mouse pairs (Fig. 6A), including 302 unique *T. muris* genes and 1,456 unique mouse genes, with each *T. muris* gene correlating with ~4.8 mouse genes on average (Table S28). Among these, TMUE_M000000039, encoding a subunit of NADH dehydrogenase (ubiquinone) involved in mitochondrial electron transport, displayed 396 expression correlations with mouse genes. However, the majority of *T. muris* genes (73.5%, 222 of 302) exhibited correlations with one or two mouse genes during infection in colonoids (Fig. 6B).

Of the 302 correlated *T. muris* genes, 19 (6.3%) were proteases, a significantly higher proportion than the 3.3% protease genes in overall *T. muris* genome (*P* = 5.7 × 10^−3^; negative binomial distribution test). Among these, serine proteases (12 genes), primarily from the S01 family, were the most frequently represented group across the three major protease classes (Fig. 6C). Notably, six of these 12 serine proteases overlapped with genes recently reported by Goulding et al. (44) as upregulated in embryonated eggs at 8 weeks compared to 6 weeks, where they were suggested to play roles in whipworm egg hatching (44). This transcriptomic overlap may indicate that some of these serine proteases are expressed across multiple early life stages and could contribute to early host–parasite interactions, and further functional studies would be needed to confirm this observation. Further refinement of correlated gene pairs using additional criteria, including signal peptide prediction, transmembrane domain analysis (allowing one or two transmembrane domains), and moderate differential expression of mouse genes (*P* ≤ 0.05), resulted in the identification of 77 *T. muris*–mouse gene pairs (Table S29).

The top 30 gene pairs with the strongest positive or negative correlations were characterized in detail, comprising 13 *T. muris* genes and 28 mouse genes, reflecting one-to-multiple gene interactions (Fig. 6D). Among these, TMUE_2000007796, encoding a potentially secreted protein of unknown function, showed a robust positive correlation with ENSMUSG00000104721, *Ifih1*, *Syap1*, and *Ubxn2a* and negative correlation with *Slc66a2, Cope*, Pdzd11, and *Pmvk* across all time points (two, 24, and 48 hpi) (Fig. 6E). In addition, three serine proteases potentially secreted by *T. muris*—TMUE_2000006856, TMUE_2000006651, and TMUE_3000012072—were identified. In a previous study, these serine proteases displayed an overall trend of increasing expression levels during *in vivo* infection stages, from egg to L1 at three hpi, L1 at 24 hpi, and L2 larvae (17). In parasitic nematodes, proteases and protease inhibitors play essential roles in food digestion and modulating the host environment (45). They are often secreted to facilitate nutrient acquisition and manipulate host tissue responses, thereby enhancing parasite survival and infection (46, 47). In our data set, TMUE_2000006856 showed negative correlations with 3-oxoacid CoA transferase 1 (*Oxct1*) and delta-like non-canonical Notch ligand 1 (*Dlk1*) and exhibited positive correlations with SS18 subunit of BAF chromatin remodeling complex (*Ss18*). *Oxct1* was significantly downregulated in the infected compared to the uninfected colonoids at two hpi (*P*_adj_ = 3.7 × 10^−3^ and log_2_ fold change = −0.67) and appeared to be involved in metabolism of lipids (*P*_adj_ = 0.019) (Tables S2 and S17). TMUE_2000006651 was positively correlated with Praja ring finger ubiquitin ligase 2 (*Pja2*) involved in protein ubiquitination and nuclear export adaptor protein (*Nmd3*). TMUE_3000012072 showed negative correlations with epithelial membrane protein 2 (*Emp2*) whose functional roles are implicated in the formation of lipid rafts and the modulation of the plasma membrane trafficking activities of integrins (48). Overall, these results outlined specific gene expression correlations between *T. muris* and the host during early infection, although further studies will be needed to evaluate and determine their functional roles during host-pathogen interaction.

## DISCUSSION

We interrogated the transcriptomes of both host and parasite during early stage (L1 larvae) of *T. muris* infection, utilizing complementary *in vivo* (B6 and STAT6KO mice) and *in vitro* (proximal colonoids) models. By integrating transcriptomic data from diverse host environments, we provided novel insights into the molecular mechanisms underlying early whipworm infection. In addition, our observations of whipworm infection in proximal colonoids aligned with and expanded on previous findings, reinforcing the potential application of intestinal organoids as an *in vitro* model system for studying host–parasite interactions during the initial stages of infection.

A recent cecaloid study demonstrated that *T. muris* L1 larvae became intracellular, burrowing through multiple IECs and preferentially infecting mitotically active cells, forming syncytial tunnels in a manner comparable to *in vivo* mouse models (17). Similarly, during early infection in proximal colonoids, we observed *T. muris* L1 larvae in close association with the epithelial layer within two hpi, with portions of the larvae appearing at different focal planes, suggestive of intracellular positioning. These larvae were preferentially found near proliferating cells (Fig. 1B through H). Furthermore, we observed that the two-dimensional monolayer colonoid culture could support whipworm infection beyond the early stages. *T. muris* larvae formed intricate syncytial tunnels and survived up to 14 days in colonoids. At nine days post-infection (dpi), some of *T. muris* larvae were positioned above the monolayer colonoid, while others were located within the same focal plane as IECs cells, indicating potential intracellular localization (Fig. S2A through C). Some larvae developed into visibly larger and thicker bodies by 14 dpi, with preliminary measurements showing increased length and width and the apparent loss of an anterior DAPI-stained structure, likely the nerve ring, observed in earlier stages. These observations suggest that the colonoid model may support not only larval persistence but also growth and early-stage morphological changes indicative of ongoing development *in vitro* (Fig. S2D through F). While further investigation is required to validate these findings, our results suggest that intestinal organoids not only offer a valuable platform for studying early-stage whipworm infection but also hold promise as a model for investigating longer-term larval development *in vitro*.

Overall, the PCA analysis revealed divergence in the 24-hour transcriptomic profiles of *in vivo* mice and colonoids (Fig. 1I). This observation is not unexpected, as the complex *in vivo* environment includes signals from immune, neuronal, and microbial components, cues that are absent in colonoids, which consist only of intestinal epithelial cells (IECs). While some pathways, such as those related to lipid metabolism, were modulated across both systems, others, particularly those involved in neurotransmitter signaling and post-translational phosphorylation, were selectively upregulated in B6 mice. Similarly, STAT6KO mice, lacking Th2 signaling, exhibited modulation of immune-related pathways, such as Fc epsilon receptor (FcεR) signaling, highlighting the influence of extrinsic cues that are absent *in vitro*. Together, these findings suggest that certain pathways influenced by systemic signals may not be fully captured in the colonoid model.

Nonetheless, *in vitro* models support the differentiation of diverse IEC subtypes observed *in vivo*, making them a simplified yet biologically relevant model to investigate the potential role of IECs in early infection dynamics (Fig. S1) (17–19). To gain further insight into the epithelial cell types modulated during early infection, we examined differentially expressed genes in the context of published single-cell RNA sequencing data from the mouse cecum (17) (Fig. S5). The analysis revealed consistent downregulation of early enterocyte markers in both *in vivo* (B6 and STAT6KO mice) and *in vitro* (colonoid) models, though the magnitude of change was more pronounced *in vivo*. In addition, expression of markers for differentiated epithelial cell types, including goblet cells (e.g., *Muc2*), enteroendocrine cells (e.g., *Pyy*), and tuft cells (e.g., *Tuba1a*), was broadly modulated across systems, with similar trends observed *in vivo* and *in vitro*, although statistical significance was not always retained.

These results suggest that while colonoids do not fully recapitulate the *in vivo* context, they offer a tractable platform for capturing epithelial cell-intrinsic transcriptional dynamics during early infection. Their utility lies in isolating epithelial responses from systemic immune and stromal influences, enabling the identification of pathways that may be initiated directly at the host-parasite interface. This makes colonoids a valuable complementary system for dissecting the molecular mechanisms that govern early infection stages.

We hypothesized that while the primary immune response likely influences later infection stages, Th2 responses in B6 mice and Th1 responses in STAT6KO mice, differences observed during the initial infection stages may act as predictors or molecular alarmins contributing to effective host defense against whipworm infection. A notable finding exclusive to B6 mice was the enrichment of upregulated genes in the acetylcholine neurotransmitter release cycle and post-translational protein phosphorylation pathways (Fig. 2D). *Vamp2* and *Snap25* are a part of the SNARE core complex, which facilitates the docking and fusion of synaptic vesicles with the plasma membrane (49). Synaptotagmin-1 (*Syt1*) binds to the SNARE complex to trigger the release of neurotransmitter/neuroendocrine substances such as acetylcholine (50). Chromogranins (*Chgb*) and secretogranins (*Scg2* and *Scg3*) are involved in the sorting, packaging, and secretion of hormones and peptide precursors (Fig. 2D) (51). These proteins are major components of the secretory granules of enteroendocrine cells (EECs), which are neuroendocrine cells found in the gastrointestinal tract (52–54). It has been suggested that EECs may play a role in the chemosensory detection of helminth infections, orchestrating immune responses in the intestine and facilitating communication between the enteric nervous system and the immune system (55–58). In addition, increased levels of chromogranin A and EEC hyperplasia have been reported in patients with inflammatory bowel disease and in animal models of colitis, indicating their regulatory roles in intestinal inflammation (59). Somatostatin (*Sst*), produced by EECs, functions as a neurotransmitter and neuromodulator in the myenteric and submucosal nerve plexus of the gut (60). In our study, *Sst* was identified as the most significantly upregulated gene exclusively in infected B6 mice and emerged as one of the top five hub genes in the PPI network, where it interacts with proteins, such as *Penk*, *Tac1*, *Grp*, *Calb2*, and *Gal*, which are associated with neuronal function and signaling (Fig. 2A and C). Somatostatin has also been shown to regulate the secretion of proinflammatory cytokines, such as IL-8 and IL-1β, from IECs, suggesting an important role in controlling mucosal inflammatory responses in the intestine (61). The neurosecretory and cholinergic responses observed in B6 mice may reflect early activation of pathways associated with the “weep and sweep” response, in which tuft cell-derived acetylcholine contributes to worm clearance (62). Tuft cells, which express choline acetyltransferase, have been shown to secrete acetylcholine into the gut lumen upon helminth infection, promoting epithelial fluid secretion and directly impairing worm fecundity (63, 64). Notably, tuft cells are considered sentinels for parasitic infections, capable of sensing luminal cues and initiating type 2 immune responses (65). In our study, while we did not directly measure tuft cell activation, we observed a significant increase in the transcriptional level of Dclk1, a marker for tuft cells, and enrichment of acetylcholine-related pathways in B6 mice. These findings suggest early neuroimmune communication that may prime the host for effective defense. Furthermore, increased tuft cell populations have been reported during helminth infections, including whipworm (17, 65, 66), further supporting the potential involvement of tuft cells in the responses observed in our study. Interestingly, genes related to neurotransmitter release and neuroendocrine secretory pathways were significantly upregulated in B6 mice but not in STAT6KO mice or colonoids (Fig. 2D). This differential response underscores the potential contribution of immune-competent pathways in B6 mice during early infection, which may be absent or less functional in the immunocompromised STAT6KO model or the simplified colonoid system, suggesting their important roles in the host’s initial response to *T. muris* infection.

One of the suppressed pathways uniquely enriched in STAT6KO mice was the Fc epsilon receptor (FcεR) signaling pathway, which is involved in IgE-mediated immune response (Fig. 3C). In the PPI network analysis, *Jun* and *Fos* emerged as top hub genes and appeared to interact with each other, forming the activator protein 1 (AP-1) complex (Fig. 3D). AP-1 synergizes with STAT6 to induce transcription of mouse germline ε transcripts, which is an essential step preceding Ig isotype switching to IgE (67). A study of human trichuriasis in a highly endemic area demonstrated a negative correlation between the level of *T. trichiura*-specific IgE antibodies and worm burden (68). Although the production and regulation of IgE are primarily governed by Th2-mediated immune responses, which are important for the host protection against the whipworm infection, the specific role of IgE in the host protection is not known (69). In addition, two cytokine genes (*IL-15* and *IL-18*) were significantly downregulated (Fig. 3D). IL-18, which interacts with Jun in the PPI network, is a pro-inflammatory cytokine important for modulating not only Th1 but also Th2 immune responses. Depending on the presence or absence of IL-12 and/or IL-15, IL-18 either induces transcription of IFN-γ (Th1-mediated response) by activating AP-1 and STAT4 or promotes differentiation of naïve T cells to Th2 cells, which produces type 2 cytokines such as IL-4 and IL-13 (70, 71). The suppression of the Fc epsilon receptor signaling pathway, coupled with the downregulation of *Il15* and *Il18*, observed exclusively in STAT6KO mice but not in B6 mice or colonoids, implicates a potential relationship between STAT6 deficiency and the functional roles of these pathways during early infection. These molecular changes may indicate its roles as markers or molecular triggers contributing to the host’s ability to mount an effective defense against whipworm infection. However, further studies are needed to clarify the functional implications of this downregulation and its impact on the host’s ability to mount effective immune responses against whipworm infection.

Comparative analysis of host transcriptional responses across *in vivo* and *in vitro* models revealed metabolism, particularly lipid metabolism, as a commonly modulated molecular mechanism during L1 *T. muris* infection. Parasitic nematodes are widely recognized as being unable to synthesize all necessary lipids *de novo* and are thus dependent on host-derived lipids, with the exception of lipid biosynthesis observed in *T. globulosa* (72, 73). In our data set, B6 mice exhibited the most significant changes in lipid metabolism-related genes, with 88 of 295 downregulated genes associated with metabolism, 41 with lipid metabolism, and 10 with the cholesterol biosynthesis pathway. For instance, genes involved in lipid metabolism and cholesterol synthesis (*Hmgcs1*, *Mvd*, *Idi1*, *Fdps*, *Fdft1*, *Lss*, *Msmo1*, *Nsdhl*, and *Cyp51*) showed reduced expression in B6 mice following L1 *T. muris* infection (Fig. 4F). Interestingly, a previous study reported transcriptional changes of the same genes during the L3-L4 larval stages of *T. suis* infection, but with different direction of transcriptional modulation (74). In our study, the suppression of lipid metabolism genes was not limited to B6 mice (Fig. 3E). Genes in lipid metabolism pathways were also downregulated in STAT6KO mice, suggesting that modulation of host metabolism may represent an intrinsic response to *T. muris* infection that is not entirely dependent on a fully functional immune system. In colonoids, we observed a dynamic regulation of *Gale*, *Fasn*, and *Mvd* (genes involved in galactose, fatty acid, and cholesterol metabolism), which were significantly upregulated at two hpi but downregulated at 24 hpi (Fig. 4F). The consistent downregulation of these genes in B6 mice suggests that metabolic modulation is likely driven, at least in part, by IECs and may be a fundamental aspect of the host’s response to whipworm infection. However, it is important to note that this experimental design represents a snapshot of gene expression at a specific time point during early infection, and these changes may not capture lipid metabolism dynamics throughout the entire infection cycle. Moreover, previous studies, such as those demonstrating upregulation of lipid metabolism genes during later stages of *T. suis* infection, highlight the complexity of host–parasite interactions over time. Thus, further research is required to clarify the causal relationships, functional significance, and temporal dynamics of these observations.

In addition to characterizing host transcriptomic profiles, we demonstrated that colonoids can be utilized to investigate diverse aspects of whipworm infection. During the *in vivo* infection study, *T. muris* L1 larvae were isolated from B6 and STAT6KO mice at 24 hpi. However, this required sacrificing eight to 10 mice per biological replicate to collect approximately 2,000 L1 larvae, and even then, the yield was insufficient to achieve robust *T. muris* transcriptomic read coverage. In several replicates, host-derived reads dominated the sequencing data, reflecting the technical challenges of isolating host-free L1 *T. muris*, which are small in size and located intracellularly at the bottom of intestinal crypts during this stage. In contrast, the use of colonoids combined with a dual-RNA sequencing approach–which circumvents the need for worm isolation–enabled the characterization of temporal transcriptomic profiles of L1 *T. muris* during early-stage infection. Although the experimental design was intended to compare *T. muris* L1 transcriptomes across *in vivo* (B6 and STAT6KO) and *in vitro* colonoid environments, substantial variation and limited L1 RNA recovery in *in vivo* samples precluded direct statistical comparisons or correlation analysis. Nonetheless, colonoids provided more consistent L1 read recovery across multiple time points, allowing for a temporal filtering and clustering strategy to uncover dynamic gene expression patterns. While this method also presented challenges due to the limited representation of worm-derived sequences due to host-dominant transcriptomes, an optimized filtering process helped mitigate this limitation by leveraging samples across multiple time points. Of note, we observed that the low proportion of *T. muris*-derived sequencing reads remained relatively stable across the infection timeline. The average mapping percentages to the *T. muris* genome were ~0.4% at two hpi, ~0.3% at 24 hpi, and ~0.6% at 48 hpi, with no consistent upward trend across replicates. Statistical comparison of read mapping rates using unpaired two-sample *t*-tests (assuming equal variance) across time points yielded *P*-values of 0.054 (2 vs. 24 hpi), 0.213 (2 vs. 48 hpi), and 0.090 (24 vs. 48 hpi). This suggests that *T. muris* transcript abundance did not substantially increase over the 48-hour period, likely due to the short infection window and initial worm input (~300 L1s per infection). Compared to the *in vivo* setting, where sampled tissue may not be spatially confined to infected sites, the colonoid model ensures a more localized and uniform infection zone. The transcriptome captured represents the most abundant *T. muris* transcripts, likely involved in host interaction and early-stage adaptation, which are especially relevant for understanding the molecular events of early infection. These findings highlight the utility of the colonoid model for enabling time-resolved analyses within a defined infection context. Future studies incorporating parasite enrichment strategies or deeper sequencing may help improve detection sensitivity and better capture transcriptional dynamics. Although not without caveats, this approach offers a promising and practical alternative to relying exclusively on *in vivo* models, particularly when aiming to reduce animal use and improve scalability. Future studies could build upon this foundation by increasing the number of worms per colonoid culture or optimizing sequencing protocols to enhance worm-specific read capture. Furthermore, the colonoid model facilitated host–parasite co-expression analyses, allowing for the identification of gene pairs concurrently regulated during infection. In our correlation analysis, we did not explicitly subtract background expression dynamics from uninfected colonoids, because the analysis was intended to capture temporal co-expression patterns between host and parasite genes during infection. While some genes may be affected by baseline temporal variation, whipworm infection may further modulate these trajectories through subtle amplification or sustained expression. To address this possibility, we examined uninfected colonoids and found that only 32 and 28 of the 1,465 host genes identified in the full correlation set (*n* = 1,611 host–parasite gene pairs) showed consistent up- and downregulation, respectively, across all time point comparisons (2 > 24 > 48 hpi or 2 < 24 < 48 hpi). In the refined correlation subset (*n* = 77), only two of the 75 unique host genes showed consistent upregulation across the infection timeline: Fth1 and Tmem140. This suggests that most correlated host genes are not simply reflecting background temporal trends.

Together, our findings provide a foundational framework for understanding the molecular interplay between *T. muris* and its host during the early phase of infection, identifying shared molecular mechanisms and potential metabolic and other molecular targets for intervention. By integrating *in vivo* and *in vitro* approaches, this study not only suggests a potential role of lipid metabolism in early whipworm infection but also demonstrates colonoids as a scalable and versatile model for future investigation aimed at identifying therapeutic strategies to combat whipworm infections in the early stages.

## MATERIALS AND METHODS

### Mice and *Trichuris muris* infection

*Trichuris muris* were maintained in susceptible mixed-sex C57BL/6N/STAT6KO mice (derived from breeding pairs of B6.129S2(c)-Stat6 (tm1Gru)/J purchased from Jackson Laboratories, Bar Harbor, ME) that were inoculated *per os* (orally) with 250 infective *T. muris* eggs. Adult *T. muris* were recovered from infected mice between 32 and 34 days post-inoculation and manually removed from the cecum and proximal colon using forceps. The adult worms were washed free of debris by settling and decanting in 50 mL tubes containing PBS (×5) followed by RPMI 1640 medium (×5) after a two-hour incubation in a 37˚C water bath. Approximately 100 adult worms were then distributed into individual 6-well culture plates containing RPMI1640 medium and maintained overnight in a 37˚C incubator with 5% CO_2_. The eggs that were released in culture overnight were isolated using a 10 mL pipette, after manual removal of the adult worms from the culture wells, washed three times in PBS by centrifugation at 200 × *g* for five minutes with removal of the supernatants, followed by resuspending the eggs in 6-well culture plates containing PBS that were maintained at room temperature for approximately 60 days. Visual inspection of the developing eggs was performed using an inverted microscope and considered infective when well-differentiated L1 were observed in the eggs. The functional infectivity of the batch of eggs was determined by visually estimating the number of infective eggs in the inoculating dose using an inverted microscope and inoculating STAT6KO mice followed by recovery of adults as above. Studies using wild-type C57BL/6N (B6) mice were derived from breeding pairs purchased from Jackson Laboratories, Bar Harbor, ME.

Mixed-sex STAT6KO or B6 mice were inoculated with approximately 300 infective eggs by oral gavage. Proximal colons of uninfected and infected mice at 24 hours post-infection (hpi) were collected for RNA isolation and sequencing. We note that a mixed population of male and female mice was used in this study without stratification by sex. Although sex-based differences in *T. muris* expulsion have been reported during chronic infection in immune-deficient mouse models, such effects are largely attributed to later-stage adaptive immune responses (37). Because our study focused exclusively on early infection (up to 48 hpi), a phase likely dominated by epithelial and innate signals, we did not expect significant sex-specific transcriptional differences at this stage. Future investigations that explicitly account for sex as a biological variable will be valuable to assess potential early differences in host responses to whipworm infection.

### Establishment and air-liquid interface (ALI) culture of mouse proximal colonoids

The mouse (proximal) colonic spheroid line was established from six-week-old female C57BL/6J mice as previously described (75). Briefly, a 1 cm segment of proximal colon was minced and treated with 2 mg/mL collagenase I solution for 10 to 20 minutes with vigorous pipetting every 10 minutes. Released crypts were collected by centrifugation, washed twice with washing medium (DMEM/F12 with HEPES, 100 units/mL penicillin, 0.1 mg/mL streptomycin, 2 mM L-glutamine, and 10% FBS), resuspended in 15 µL of Matrigel, and plated onto a 24-well tissue culture plate with 500 µL of 50% L-WRN conditioned medium.

Mouse proximal colonic spheroids were maintained and passaged in 50% L-WRN conditioned medium (CM) supplemented with 10 µM Rho kinase (ROCK) inhibitor (Y-27632) (50% CM) as previously described (18, 75). Then, spheroids were dissected into single cells using TrypLE digestion for five minutes at 37°C, and 200,000 cells suspended in 100 µL of 50% CM were seeded onto each Transwell pre-coated with 5% Matrigel for 10 minutes. Medium in the upper chamber of the Transwell was removed after two to three days to create an air-liquid interface (ALI). Cells were grown and differentiated in ALI culture with 650 µL of 50% CM in the bottom chamber for an additional 21 days. The 50% CM was changed every two days.

### *In vitro* hatching of *T. muris* eggs

Infective *T. muris* eggs were stored at 4°C in PBS and then incubated with 1.8% sodium hypochlorite at 37°C with 5% CO_2_ for 1.5–2 hours. Eggs were washed twice with PBS and once with RPMI 1640 supplemented with 20% FBS, 100 units/mL penicillin, 0.1 mg/mL streptomycin, and 1× antibiotic-antimycotic (RPMI 1640 medium) followed by incubation with RPMI 1640 media at 37˚C with 5% CO_2_ for three to five days until the L1 hatched.

### L1 *T. muris* infection in mouse proximal colonoids in ALI culture

Approximately 300 *in vitro*-hatched L1 *T. muris* larvae were collected by centrifugation at 720 rcf for 5–10 minutes and suspended in 50 µL of 50% CM. The L1 suspension was introduced to the apical surface of differentiated proximal colonoids in ALI culture at 21 days. Infections were maintained for 2, 24, and 48 hours at 37°C with 5% CO_2_.

### Immunofluorescence staining of mouse colonoids

ALI cultured colonoid cells were fixed in 4% paraformaldehyde (PFA), methanol-free (Thermo Fisher, AA47392-9L) for 30 minutes at room temperature (RT) and washed three times with Dulbecco’s phosphate-buffered saline (DPBS) without calcium and magnesium (Thermo Fisher, 14190250). Colonoid cells were permeabilized and blocked with DPBS containing 5% goat serum (Thermo Fisher, 16-210-064) and 0.25% Triton X-100 (Sigma-Aldrich, T8787) for one hour at RT and then treated with primary antibodies diluted in DPBS containing 1% goat serum and 0.25% Triton X-100 overnight at 4˚C: rabbit polyclonal anti-MUC2 (1:100, Thermo Fisher, PA5-119291), rabbit polyclonal anti-Chromogranin A (1:100, Thermo Fisher, PA5-85952), rabbit polyclonal anti-DCLK1 (1:100, Thermo Fisher, PA5-20908), rabbit polyclonal anti-Ki-67 (1:100, Thermo Fisher, PA5-143611), rabbit monoclonal anti-villin (1:100, Thermo Fisher, MA5-16408), Ulex Europaeus agglutinin I (UEA I), and rhodamine (1:100, Vector Laboratories, RL-1062-2). Cells were washed with DPBS three times and incubated with secondary antibody goat anti-rabbit IgG Alexa Fluor Plus 488 (1:1,000, Thermo Fisher, A32731TR), phalloidin Alexa Fluor 594 (1:400, Thermo Fisher, A12381), and 4′,6-diamidino-2-phenylindole dihydrochloride (DAPI ready-made solution, 1:1000, Sigma-Aldrich, MBD0015-1ML) for one hour at RT. After three washes with DPBS, cells in Transwell membranes were mounted on slides using ProLong Gold Antifade reagent (Thermo Fisher, P10144). Fluorescence images were captured using a Nikon ECLIPSE Ti2 inverted confocal microscope.

### Preparation of samples for RNA-seq and production of RNA-seq data

For *in vivo* analysis, approximately 1 cm of proximal colon tissue was collected from each mouse (infected or uninfected). Total RNA yield ranged from 30 µg to 92 µg per sample, and approximately 50 µg of RNA was typically used for cDNA library preparation.

For *in vitro* experiments, RNA was extracted from colonoid monolayers grown on 6.5 mm transwell inserts (Corning 3470) co-cultured with and without ~300 L1 larvae. Infected colonoids and co-cultured L1 larvae were lysed together, and RNA was purified from the combined lysate to obtain transcriptomic profiles from both host and parasite. RNA yield from each insert was approximately 1 µg of total RNA, which was used in full for RNA-seq library preparation.

In both cases, RNA was extracted using Trizol (Invitrogen) following homogenization with 1.5 mm zirconium beads (Benchmark Scientific, D1032-15) with a BeadBug6 (Benchmark Scientific, D1036, Sayreville, NJ, USA). cDNA libraries were prepared using poly(A) enrichment and sequenced on the Illumina NovaSeq S4 platform (paired-end 150 bp reads). For each condition, three to four biological replicates were prepared.

### Preparation of RNA samples and production of RNA sequencing

Sequencing reads were aligned to the mouse reference genome (GRCm39) and the *T. muris* reference genome (Trichuris_muris.TMUE3.0) retrieved from the Ensembl database (76). The alignment was performed using RSEM (version 1.3.1) to estimate the expression abundances of transcripts and genes (77). The raw RNA-seq read files (fastq) are accessible on the NCBI Sequence Read Archive (78), with accessions per sample provided in Table S1.

### Mouse transcriptomic analysis

For host (mouse) transcriptomic analysis, DESeq2 (version 1.34.0 [29]) was used to perform differential expression analysis between infected samples and uninfected controls. Significant DEGs were determined using a threshold of *P*_adj_ ≤0.05 and an absolute fold change ≥1.3. Functional enrichment pathway analysis was performed using ConsensusPathDB with Reactome pathways (79). All Ensembl gene IDs were provided as the background gene list with default setting. Significance was determined with a threshold cutoff of *P*_adj_ ≤0.05. In addition, Reactome pathway annotation network analysis was performed using ClueGo (version 2.5.10 [80]) and CluePedia (version 1.5.10 [81]) plugins in Cytoscape (version 3.10.1 [82]) with a Bonferroni step-down corrected *P*-value cutoff ≤0.1. The STRING database (version 12.0 [30]) was used to identify protein-protein interaction (PPI) networks of DEGs with a confidence cutoff of 0.7, followed by visualization with Cytoscape. Rates of alternative splicing (AS) exons were estimated as a percent spliced in (PSI), which is a fraction of mRNA including the AS exons from total mRNA, using rMATs with the RNA-seq reads (83). PSI levels were compared between infected and uninfected conditions. Four types of AS were considered: exon skipping (ES), alternative 3′ splice site (A3SS), alternative 5′ splice site (A5SS), and intron retention (IR). Significantly differential AS exons were defined as those with *P*_adj_ ≤0.1 and differential PSI ≥ 0.05 (84).

### *T. muris* transcriptomic analysis

For transcriptomic analysis of L1 *T. muris*, genes with expression detection (FPKM > 0) in at least two of the three biological replicates at any of the three infection time points (two, 24, and 48 hpi) were selected for analysis to reduce the chance of detecting spurious gene expression due to low mapping rates. Further prioritization included genes with a maximum absolute log_2_ fold change ≥0.5 among the comparisons (2 vs. 24 hpi, 2 vs. 48 hpi, and 24 vs. 48 hpi) to ensure at least moderate differential expression. Average FPKM values were used as input for Mfuzz soft clustering (40) to identify temporal gene expression patterns. Mfuzz clustering was repeated 10 times to estimate cluster stability with the number of clusters (*c* = 6) and the fuzzifier (*m* = 2.71). Only genes assigned to the same cluster after 10 iterations were included in the final analysis.

Functional annotations for *T. muris* genes were assigned using annotations from InterProScan v5.59-91 (85), GhostKOALA v2.2 (86), Pannzer2 (87), sma3s v2 (88), SignalP v4.1 (89), and tmhmm v2.0c (90). MEROPS (91) was used to identify proteases and protease inhibitors with NCBI blastp v2.7.1+ (92) with *e*-value cutoff of 1 × 10^−4^. The best bi-directional hits were identified based on protein alignment between the longest isoforms per gene of *T. muris* and those of human (GRCh38.109) and *C. elegans* (PRJNA13758.WBPS18) using Diamond blastp (v2.0.6.144) (93). Alignments were performed in both directions: first using *T. muris* proteins as queries against the human or *C. elegans* proteomes, and then using the human or *C. elegans* proteins as queries against the *T. muris* proteome. A local script was used to compile reciprocal best hits, defined as top-scoring protein pairs that were mutual best matches in both search directions. For *C. elegans*, gene symbols and descriptions were harvested from the BioMart (94) instance hosted at WormBase Parasite (95) and used to annotate the results. Enriched KEGG pathways of genes assigned to each cluster were identified using WebGestaltR (96) (minimum two genes per pathway, *P* ≤ 0.05 threshold for significance).

### Correlation analysis of *T. muris* and mouse transcriptome

To identify pairs of genes showing expression correlation between whipworm and host, *T. muris* and mouse genes with FPKM >0 in all infected colonoid samples (three biological replicates across three infection time points) were selected. Pearson correlation coefficients (*r*) were calculated using log_10_-transformed FPKM values of the selected genes from *T. muris* and mouse, followed by choosing pairs of genes with a high correlation (*r* ≥ 0.95). In addition, to prioritize gene pairs potentially related to molecular interactions between L1 *T. muris* and mouse, mouse genes showing differential expression between infected and uninfected colonoids were selected with a *P*-value cutoff ≤0.05. *T. muris* genes encoding potentially secreted proteins were selected based on the presence of a predicted signal peptide and having ≤2 predicted transmembrane domains.

## Data Availability

NCBI SRA accession numbers for RNA sequencing data sets used in this study are available in Table S1 and are saved under BioProject PRJNA1145400.

## SUPPLEMENTAL MATERIAL

Supplemental Material mbio.02176-25-s0001.pdf Supplemental table captions and Figures S1-S5.

Supplemental Tables mbio.02176-25-s0002.xlsx Tables S1 to S29.

ASM does not own the copyrights to Supplemental Material that may be linked to, or accessed through, an article. The authors have granted ASM a non-exclusive, world-wide license to publish the Supplemental Material files. Please contact the corresponding author directly for reuse.
