# Supplementary material for: Transcriptional responses of mouse proximal colon and colonoids during early whipworm infection
Source: mBio. 2025 Sep 11;16(10):e02176-25. doi: 10.1128/mbio.02176-25 (PMC12505908; doi:10.1128/mbio.02176-25)
Supplement: Supplemental Material — Supplemental table captions and Figures S1-S5. [file mbio.02176-25-s0001.pdf]

SUPPLEMENTAL MATERIAL

Transcriptional responses of mouse proximal colon and colonoids during early whipworm infection

Hyeim Jung[1], Joseph F. Urban Jr.[2], Bruce A. Rosa[1] and Makedonka Mitreva[1, 3*]

[1] Department of Internal Medicine, Washington University School of Medicine, Saint Louis, MO, USA

[2] United States Department of Agriculture, Agricultural Research Service, Beltsville Agricultural Research Center, Animal Parasite Diseases Laboratory and Beltsville Human Nutrition Research Center, Diet, Genomics and Immunology Laboratory, Beltsville, MD, USA

[3] McDonnell Genome Institute, Washington University School of Medicine, St. Louis, MO, USA

*Correspondence: Dr. Makedonka Mitreva, mmitreva@wustl.edu

Supplemental Tables

Table S1 RNA-seq sample metadata

Table S2 A summary of RSEM and DESeq2 results of mouse samples

Table S3 Significantly enriched Reactome pathways (ConsensusPathDB) in up-regulated genes in infected B6 mice (24hpi)

Table S4 Significantly enriched Reactome pathways (ClueuGo/CluePedia) in up-regulated genes in infected B6 mice (24hpi)

Table S5 PPI network interactions (StringDB) in up-regulated genes in infected B6 mice (24hpi)

Table S6 PPI network node degrees (StringDB) in up-regulated genes in infected B6 mice (24hpi)

Table S7 Significantly enriched Reactome pathways (ConsensusPathDB) in down-regulated genes in infected B6 mice (24hpi)

Table S8 Significantly enriched Reactome pathways (ClueuGo/CluePedia) in down-regulated genes in infected B6 mice (24hpi)

Table S9 PPI network interactions (StringDB) in down-regulated genes in infected B6 mice (24hpi)

Table S10 PPI network node degrees (StringDB) in down-regulated genes in infected B6 mice (24hpi)

Table S11 Significantly enriched Reactome pathways (ConsensusPathDB) in up-regulated genes in infected STAT6KO mice (24hpi)

Table S12 PPI network interactions (StringDB) in up-regulated genes in infected STAT6KO mice (24hpi)

Table S13 PPI network node degrees (StringDB) in up-regulated genes in infected STAT6KO mice (24hpi)

Table S14 Significantly enriched Reactome pathways (ConsensusPathDB) in down-regulated genes in infected STAT6KO mice (24hpi)

Table S15 PPI network interactions (StringDB) in down-regulated genes in infected STAT6KO mice (24hpi)

Table S16 PPI network node degrees (StringDB) in down-regulated genes in infected STAT6KO mice (24hpi)

Table S17 Significantly enriched Reactome pathways (ConsensusPathDB) in up- and down-regulated genes in infected colonoids (2hpi)

Table S18 Significantly enriched Reactome pathways (ConsensusPathDB) in up- and down-regulated genes in infected colonoids (24hpi)

Table S19 List of differential exon skipping exons

Table S20 List of differential intron retention exons

Table S21 List of differential alternative 3' splice site exons

Table S22 List of differential alternative 5' splice site exons

Table S23 List of differential mutually exclusive splice exons

Table S24 Significantly enriched Reactome pathway in differential splicing genes

Table S25 A summary of RSEM results of *T. muris* samples

Table S26 A result summary of soft-clustering (Mfuzz) of infected colonoids (2, 24, and 48 hpi)

Table S27 Enriched KEGG pathways of T. muris genes in Mfuzz clusters

Table S28 1,611 gene pairs having a strong correlation between mouse and L1 *T. muris* (Pearson correlation coefficient $| r | \geq 0.95$)

Table S29 77 gene pairs having a strong correlation between mouse and L1 *T. muris* and associated with potentially secreted *T. muris* secreted proteins

Supplemental Figures

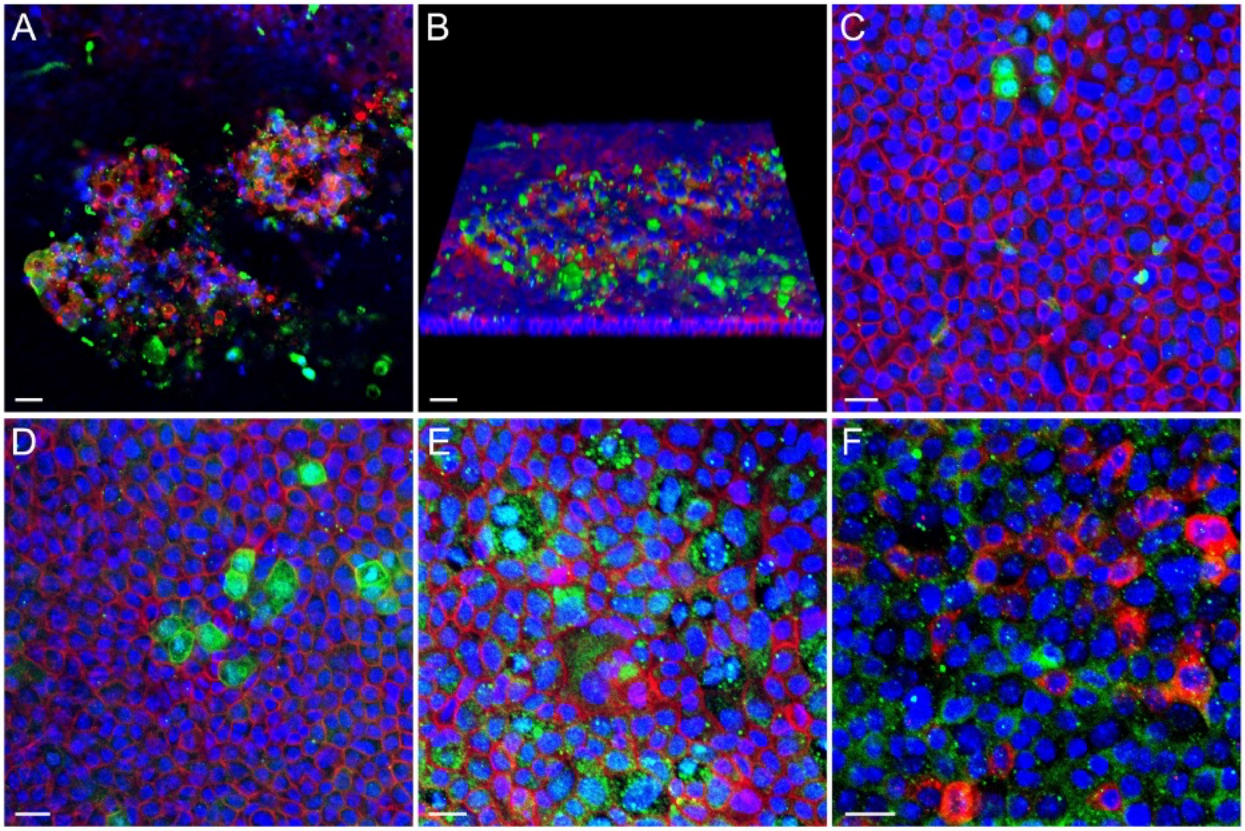

FIG S1 Representative confocal immunofluorescence images of proximal colonoids differentiated with 21 days of air-liquid interface (ALI) culture. (A,B) Monolayer culture stained with DAPI (nuclei of mouse IECs, blue), phalloidin (F-actin, red), and Ki-67 (proliferating cells, green). (C) Monolayer culture stained with DAPI, phalloidin, and Dclk1 (tuft cells, green). (D) Monolayer culture stained with DAPI, phalloidin, and chromogranin A (ChgA, enteroendocrine cells, green). (E) Monolayer culture stained with DAPI, phalloidin, and Muc2 (goblet cells, green). (F) Monolayer culture stained with DAPI, UEA I (goblet cells, red), and villin (brush border, green). Scale bars 20 μm.

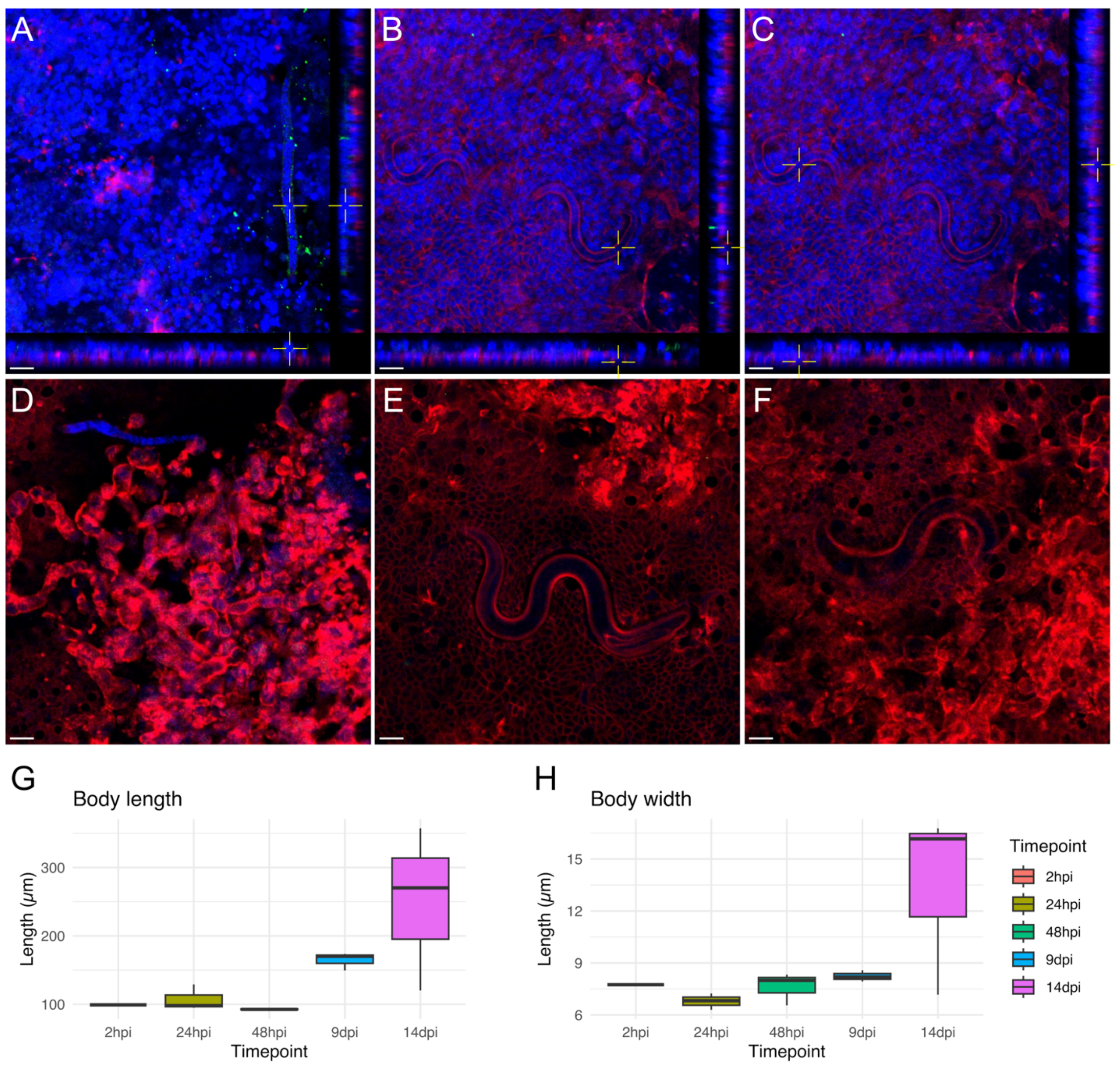

FIG S2 Representative confocal immunofluorescence images of proximal colonoids infected with L1 *T. muris* at 9- and 14-days post infection (dpi). (A-C) Colonoids infected with L1 *T. muris* at 9 dpi stained with DAPI (nuclei of mouse IECs, blue), phalloidin (F-actin, red), and Ki-67 (proliferating cells, green). Yellow crosshairs indicate focal positions of the intracellular *T. muris* larvae at different depths within the colonoid monolayer. Scale bars 20 μm. (D-F) Colonoids infected with L1 *T. muris* at 14 dpi stained with DAPI and, phalloidin. Scale bars 20 μm. (G-H) Summary of *T. muris* larval length and width measurements at 2 hpi to 14 dpi, based on confocal images. Each worm was measured three times for length and seven times for width to

improve measurement accuracy. Measurements were obtained from three biological replicates at all timepoints, except for 2 hpi, which included one replicate.

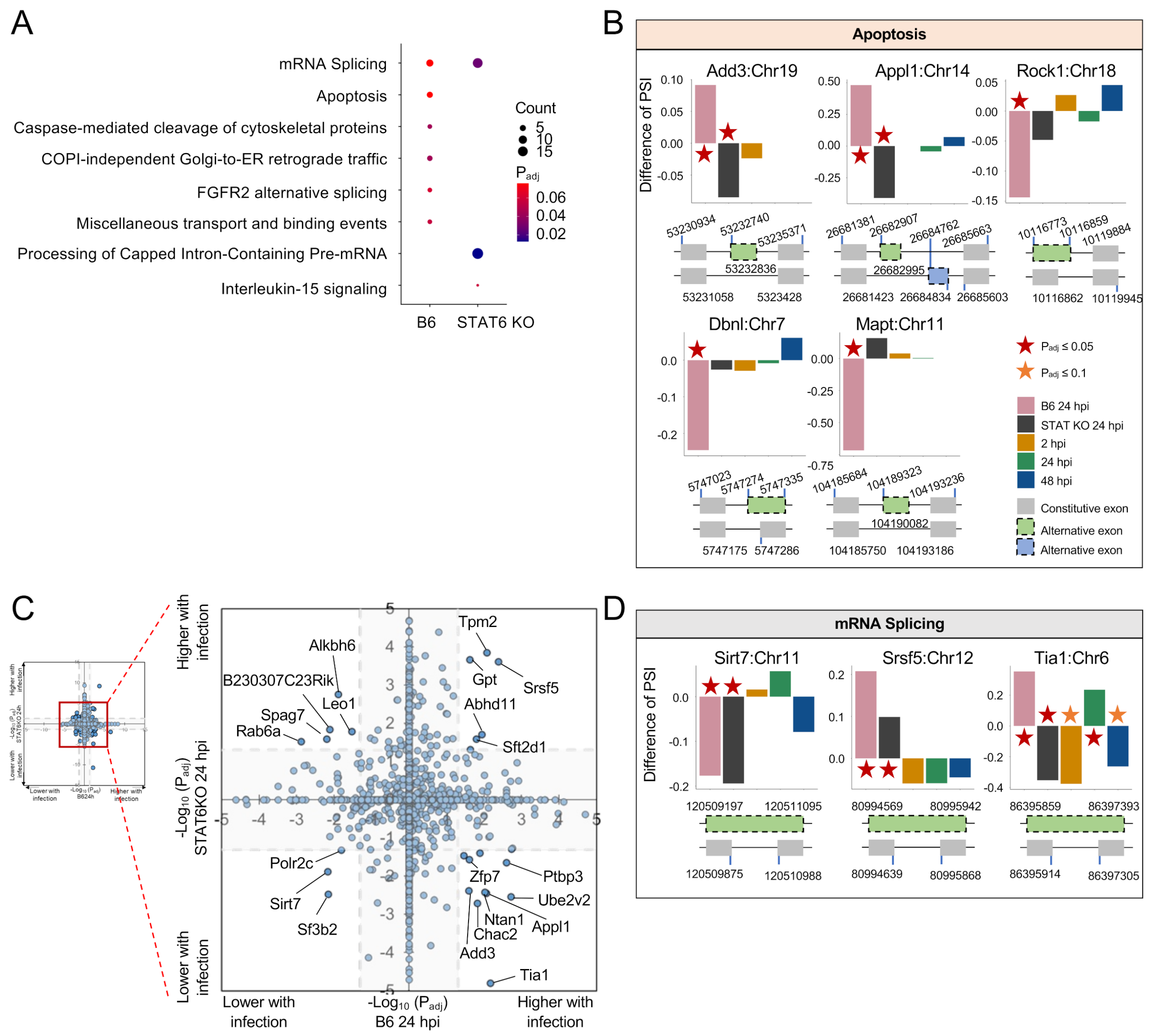

FIG S3 Alternative splicing (AS) exon analysis in B6 and STAT6 KO mice at 24 hpi. (A) Significantly enriched Reactome pathways of AS genes in infected B6 and STAT6 KO mice compared to uninfected controls at 24 hpi. The P_{adj} (q-value) for each pathway is represented by the color, and the number of significant AS genes from each pathway is represented by the dot size. (B) Bar plots with difference PSI of AS exons of genes in apoptosis pathway. Statistical significance of differential splicing (P_{adj}) between infected and uninfected mice is indicated by stars: red ($P_{adj} \leq 0.05$) and orange ($0.05 < P_{adj} \leq 0.1$). Both are considered significant in this analysis, with dual-color coding used to reflect different levels of significance within the defined

threshold range. (C) Scatter plot of $-\log_{10}(P_{adj})$ of differential AS exons of B6 and STAT6KO mice at 24 hpi. Significantly spliced-in AS exons were represented as positive $\log_{10}(P_{adj})$ values while significantly spliced-out AS exons as negative $\log_{10}(P_{adj})$ values. (D) Bar plots with difference PSI of AS exons of genes within mRNA splicing pathway. Statistical significance of differential splicing (P_{adj}) between infected and uninfected mice is indicated by stars: red ($P_{adj} \leq 0.05$) and orange ($0.05 < P_{adj} \leq 0.1$). Both are considered significant in this analysis, with dual-color coding used to reflect different levels of significance within the defined threshold range.

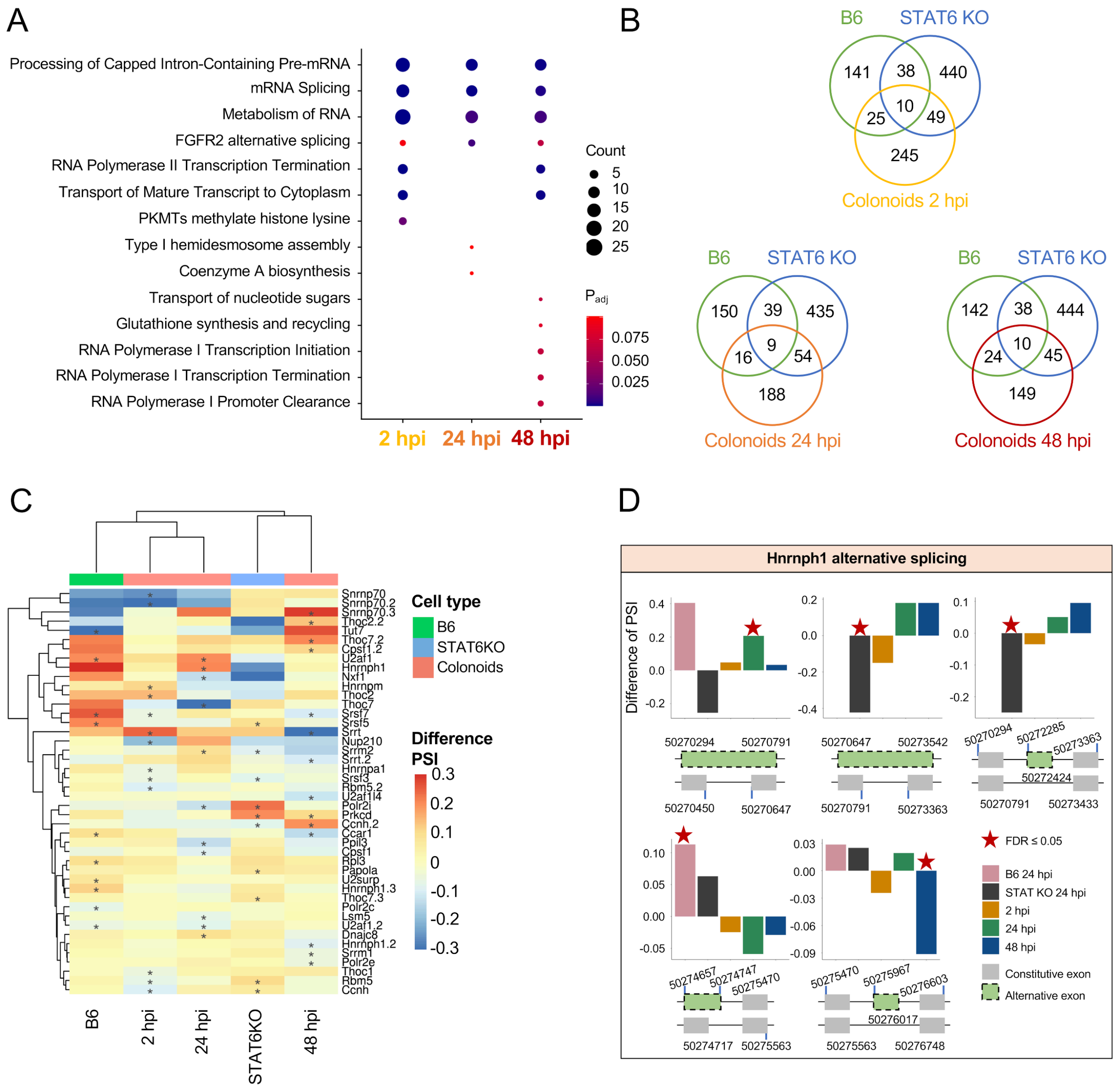

FIG S4 Alternative splicing exon analysis in colonoids at 2, 24, and 48 hpi. (A) A three-way Venn diagram of common AS genes from B6 mice at 24 hpi, STAT6KO mice at 24 hpi, and colonoids at 2, 24, and 48 hpi. (B) Significantly enriched Reactome pathways of AS genes in infected colonoids compared to uninfected controls at 2, 24, 48 hpi. The P_{adj} (q-value) for each pathway is represented by the color, and the number of significant AS genes from each pathway is represented by the dot size. (C) A heatmap and clustering based on difference PSI (percent spliced in) of AS exons of B6 mice at 24 hpi, STAT6KO mice at 24 hpi, and colonoids at 2, 24,

and 48 hpi. Statistical significance (P_{adj}) in differential AS exon between infected and uninfected mice is highlighted with an asterisk. (D) Bar plots with difference PSI of AS exons of *Hnrnph1*. Statistical significance (P_{adj}) in differential PSI between infected and uninfected mice is highlighted with a red star ($P_{adj} \leq 0.05$).

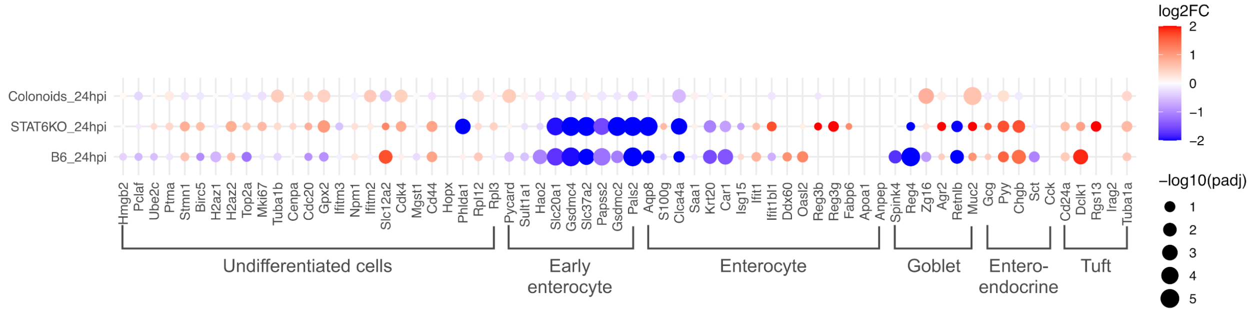

FIG S5 Cell-type marker expression in bulk RNA-seq datasets during early *T. muris* infection. Dot plot showing the differential expression of selected intestinal epithelial cell (IEC) subtype markers derived from a published single-cell RNA-seq study of the mouse cecum (17). Each row represents a bulk RNA-seq dataset from a specific condition (B6, STAT6KO, or colonoids at 24 hpi). Columns represent cell-type-specific markers, grouped by IEC subtype. Dot color indicates the $\log_2$ fold change ($\log_2$ FC) in expression between infected and uninfected samples, and dot size reflects statistical significance (-$\log_{10}$ adjusted p-value).
